# *Cynara cardunculus* subsp. *cardunculus* (Wild Artichoke) Extract: Antimicrobial Activity and Cytotoxicity, Apoptosis Induction, and Chemosensitization in Colon Cancer Cells

**DOI:** 10.3390/biology15060475

**Published:** 2026-03-15

**Authors:** Simone Bianchi, Rosaria Acquaviva, Claudia Di Giacomo, Barbara Tomasello, Francesco Pappalardo, Alessandra Pino, Irina Naletova, Donata Condorelli, Alfonsina La Mantia, Ignazio Barbagallo, Cinzia Randazzo, Giuseppe Antonio Malfa

**Affiliations:** 1 Department of Drug and Health Science, University of Catania, Via Valdisavoia, 5, 95123 Catania, Italy; simone.bianchi@unict.it (S.B.); racquavi@unict.it (R.A.); btomase@unict.it (B.T.); francesco.pap82@gmail.com (F.P.); donatacondorelli@libero.it (D.C.); alfy.lamantia@gmail.com (A.L.M.); 2 Research Centre on Nutraceuticals and Health Products (CERNUT), University of Catania, Viale A. Doria 6, 95125 Catania, Italy; alessandra.pino@unict.it (A.P.); cinzia.randazzo@unict.it (C.R.); 3 PLANTA/Center for Research, Documentation and Training, Via Serraglio Vecchio 28, 90123 Palermo, Italy; 4 Department of Agriculture, Food and Environment, University of Catania, Via Santa Sofia 100, 95123 Catania, Italy; 5 Institute of Crystallography, Research National Council, Via Paolo Gaifami, 18, 95126 Catania, Italy; irina.naletova@cnr.it; 6 Department of Biomedical and Biotechnological Sciences, University of Catania, Via Santa Sofia 97, 95124 Catania, Italy; ignazio.barbagallo@unict.it

**Keywords:** colorectal cancer, CaCo-2 cells, HFF-1, 5-fluorouracil, phenolic acids, flavonoids, chlorogenic acid, cynarine, *Enterococcus faecalis*, redox homeostasis

## Abstract

Colorectal cancer (CRC) is still a leading cause of cancer-related death worldwide, and often, conventional chemotherapeutics exhibit limited efficacy. The hydroalcoholic leaf extract of *Cynara cardunculus* subsp. *cardunculus* (wild artichoke) was investigated for its anticancer potential in CRC and effects on enteric pathogens. Nine phenolic compounds were identified by high-performance liquid chromatography with diode-array detection (HPLC-DAD), and spectrophotometric analyses were applied for total phenolic (TPC: 178.33 mg GAE/g) and total flavonoid (TFC: 52.21 mg CE/g) content quantification. The extract exhibited good antioxidant activity on DPPH (IC_50_: 21.35 μg/mL), ^−•^O_2_ (IC_50_: 1.56 μg/mL), and H_2_O_2_ (IC_50_: 314.73 μg/mL) and was found to inhibit the growth of pathogenic enteric bacteria, with *Enterococcus faecalis* and *Staphylococcus aureus* being the most sensitive. In CaCo-2 CRC cells, the extract induced a concentration-dependent cytotoxicity (IC_50_: 13.07 μg/mL at 24 h) through increased production of reactive oxygen species (ROS), upregulation of Nrf2, and induction of apoptosis, as evidenced by elevated p53, Bax, cytochrome c, and caspase-3 levels. No necrosis, measured by lactate dehydrogenase (LDH) release, or toxicity to HFF-1 normal fibroblasts was observed at concentrations up to 50 μg/mL. Additionally, CCE demonstrated synergistic effects with 5-FU (combination index < 0.8). This evidence suggests that CCE exhibits selective antitumor activity and chemosensitizing properties, supporting its possible development as an adjunctive agent in CRC therapy.

## Simple Summary

Colorectal cancer is a major health problem worldwide and one of the top causes of cancer deaths. Treatments like chemotherapy are the gold standard, but they have limitations, especially in advanced cases, where cancer cells can become resistant to the treatment. Moreover, these approaches can harm healthy cells, leading to unpleasant side effects. To find a possible solution, we tested an extract from wild artichoke leaves, a plant from the Mediterranean region that contains health-promoting substances called polyphenols, to explore whether it could kill isolated cancer cells without affecting normal cells. Interestingly, our results showed that the extract can disrupt the inner balance of cancer cells, leading to apoptosis. Notably, normal cells were unaffected by the treatment, except at a concentration of 100 μg/mL. Additionally, we also observed that when combined with the commonly used chemotherapeutic 5-fluorouracil, the extract enhances the drug efficacy reducing the concentration necessary to exert its activity by about ten times (IC_50_ 5-FU alone 5.46 μg/mL; IC_50_ 0.5 μg/mL in combination with CCE 5.88 μg/mL). Moreover, we also observed that the extract inhibits the growth of enteric pathogens. These results suggest that wild artichoke extract could help make cancer treatments safer and more effective by improving chemotherapy and reducing side effects, offering a new natural option to fight this serious disease.

## 1. Introduction

Among the most prevalent malignancies, colorectal cancer (CRC) is still a leading cause of cancer-related death and a major public health challenge [1,2,3]. By 2035, global CRC mortality is projected to rise by 60–70% [4]. Although most CRC cases and deaths occur in individuals over 70 years of age, the incidence among younger adults is rising. CRC incidence and mortality rates differ according to age, geographic region, and development index [1,4]. Established risk factors include smoking, physical inactivity, obesity, high consumption of red or processed meat, low dietary fiber intake, inflammatory bowel disease, and alcohol use [5,6,7,8,9]. The combination of these modifiable lifestyle components increases CRC risk [10], along with alterations in the gut microbiome and diet-associated epigenetic modifications [11,12]. Familial adenomatous polyposis or a family history of CRC further raises the risk [13,14]. Protective factors include antioxidants, non-steroidal anti-inflammatory drugs, and routine physical activity [4]. CRC develops through diverse molecular pathways, such as mutations in tumor suppressor genes or oncogenes, dysregulation of the cell cycle and apoptosis, and disrupted cell-to-cell interactions [15,16]. Aberrant methylation patterns also have an essential role. Roughly 20% of CRC cases are metastatic at diagnosis, with a five-year survival rate of just 15%, yet earlier stages offer a much better prognosis, underscoring the value of timely detection [17]. Management of metastatic CRC is still difficult; however, targeted therapies in combination with chemotherapy, such as checkpoint inhibitors (nivolumab, pembrolizumab, ipilimumab) [18], anti-EGFR agents (cetuximab, panitumumab), anti-HER-2 drugs (trastuzumab, pertuzumab, lapatinib) [19,20], BRAF inhibitors (encorafenib) [21], and KRAS G12C inhibitors (sotorasib and adagrasib) [22], have contributed to increased survival outcomes.

In addition to specific clinical strategies, recent research has progressively examined natural compounds with established antitumor properties, including polyphenols, flavonoids, alkaloids, and terpenoids [23,24]. These active phytochemicals modulate key oncogenic signaling pathways, inhibit proliferation in tumor cells, induce apoptosis, and regulate immune responses, as demonstrated for dietary polyphenols and flavonoids in colorectal and colon cancer models [23]. Preclinical and emerging clinical evidence suggests that natural compounds can act synergistically with conventional chemotherapeutic agents and targeted therapies, as a result improving treatment efficacy, overcoming drug resistance, and reducing treatment-associated toxicity [25].

*Cynara cardunculus* subsp. *cardunculus* L. [*Cynara cardunculus* L. var. *sylvestris* (Lam.) Fiori], commonly known as wild artichoke, is recognized as a significant source of phenolic-rich phytochemicals. This spiny species is closely related to the cultivated globe artichoke [*C. scolymus* L., *C. cardunculus* var. *scolymus* (L.) Fiori, *C. cardunculus* subsp. *scolymus* (L.) Hegi] and is widely distributed across the Mediterranean basin [26]. Wild artichoke has traditionally been used for its nutritional and medicinal properties, particularly for supporting digestive and hepatic functions. The aerial parts contain high levels of phenolic compounds and flavonoids, which are linked to various beneficial biological activities [27,28].

Given the increasing interest in phenolic compounds as potential anticancer agents, this study analyzed the hydroalcoholic leaf extract of wild *Cynara cardunculus* subsp. *cardunculus* (CCE) collected from the southern coast of Sicily, Italy. The extract was characterized for its phenolic composition and antioxidant capacity using cell-free assays, followed by evaluation of its safety in HFF-1 normal healthy cells and cytotoxicity in Caco-2 cells, an in vitro model of CRC. Its effects on tumor cellular redox homeostasis and cell death were assessed, as well as its potential as a chemosensitizer. Specifically, alterations in intracellular redox balance (ROS and GSH) and modulation of key molecular markers involved in the oxidative stress response (Nrf2) and apoptosis (p53, Bax, Cytochrome C, and caspase 3) were examined. Finally, the potential chemosensitizing effect in combination with 5-fluorouracil (5FU), a standard chemotherapeutic agent in CRC treatment, and the antimicrobial activity against major intestinal pathogens were investigated.

## 2. Materials and Methods

### 2.1. Reagents and Chemicals

Solvents suitable for HPLC analysis, including UHPLC-grade water (18 mW), were provided by Carlo Erbâ^®^ (Milan, Italy). Dimethyl sulfoxide (DMSO), 2′,7′-dichlorofluorescein diacetate (DCFH-DA), and 2,2-diphenyl-1-picrylhydrazyl (DPPH) were purchased from VWR (Milan, Italy). Unless otherwise specified, all remaining reagents were supplied by Sigma-Aldrich (Milan, Italy). Materials and culture media used for cell-based experiments were obtained from Thermo Fisher Scientific (Milan, Italy), except where otherwise specified.

### 2.2. Plant Material and Extraction Procedure

**Figure 1:**
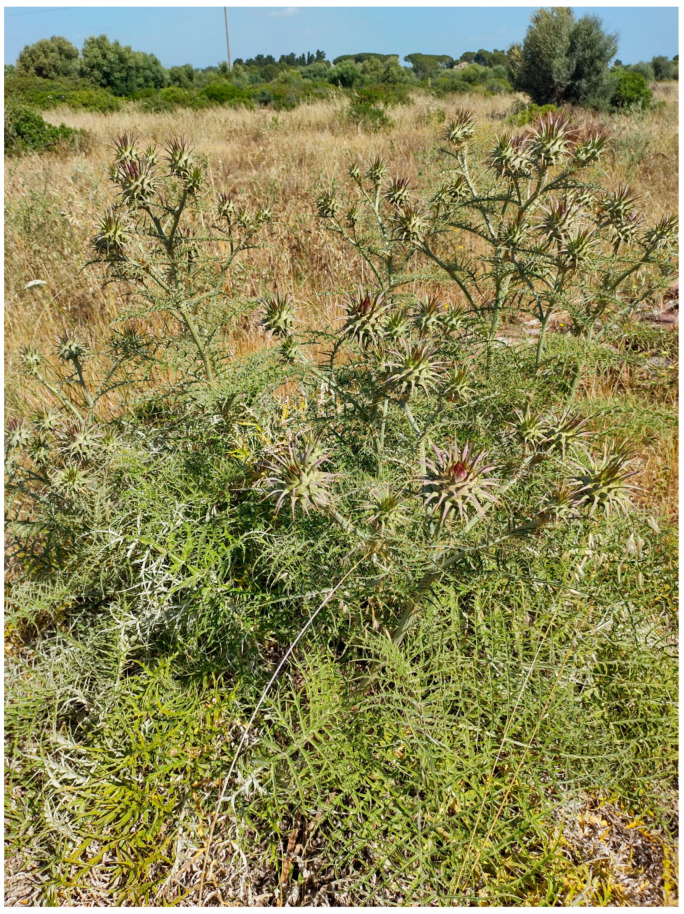
Flowering specimen of *Cynara cardunculus* subsp. *cardunculus* L. at the site of harvest in Syracuse, Italy.

*Cynara cardunculus* subsp. *cardunculus* leaves (Figure 1) were collected from the seacoast area of Syracuse, Sicily, Italy, at the end of April 2024, and authenticated by the pharmaceutical botanist G.A.M. The material was collected from different adult plants of more than 24 months and a height of about 100 cm. A voucher specimen (No. 04/24) was deposited in the herbarium of the Department of Drug and Health Sciences, University of Catania. Following collection, the leaves were washed, wiped, and stored at −80 °C to maintain the stability of the bioactive compounds. Frozen, crushed plant material (100 g) was extracted with a hydroalcoholic solution (50:50) at 80 °C for 1 h, using a 1:10 (plant:solvent) ratio. The extraction was performed three times, and the combined solutions were filtered (Whatman filter paper Grade 4) and evaporated to dryness using a rotary evaporator (40 °C), yielding approximately 5.25 g of dry extract.

### 2.3. HPLC-DAD Analysis

High-pressure liquid chromatography (HPLC) was employed to assess the polyphenolic fingerprinting of the extract as previously described [28]. HPLC-DAD analyses were performed on a Shimadzu LC-20 system (Kyoto, Japan) equipped with a diode-array detector (DAD) and a 150 × 4.6 mm i.d., 2.7 μm Ascentis Express C18 column (Supelco, Darmstadt, Germany). The mobile phases consisted of H_2_O/H_3_PO_4_ (99:1, solvent A) and MeOH/ACN/H_3_PO_4_ (49.5:49.5:1, solvent B). The gradient program was as follows: solvent A at 95% decreasing to 77% over 34 min, held at 77% for 3 min, then reduced to 74% at 60 min, 60% at 85 min, 20% at 90 min, and 0% at 92 min, with a total run time of 105 min. The injection volume was of 5 μL, the flow rate 1 mL/min and the column temperature at 25 °C. Chromatogram profiles were recorded from 190 to 500 nm and monitored at 280 and 330 nm ± 2 nm. Secondary metabolites were identified by comparing their retention time and the UV–Vis absorption spectrum with those of standard compounds registered in our internal library (see Supplementary Materials).

### 2.4. Determination of Total Flavonoid and Total Phenolic Content

CCE total phenolic content (TPC) and total flavonoid content (TFC) were evaluated, respectively, using the Folin–Ciocalteu and the aluminum chloride methods [29]. Results were compared with calibration curves prepared with a known quantity of gallic acid (TPC) or catechin (TFC) and expressed as milligrams of gallic acid equivalents (eq.) per gram of extract (mg GAE/g extract) or milligrams of catechin eq. per gram of extract (mg CE/g extract). All data represent the mean of three independent determinations.

### 2.5. DPPH^•^ Assay

The DPPH^•^ radical scavenging assay was carried out as described by Luca et al. [29]. Briefly, different CCE concentrations have been mixed with 86 μM DPPH^•^ in a final volume of 1 mL. Ethanol has been used as solvent for the reaction mix, which was incubated for 10 min, at room temperature, in the dark, before measuring the absorbance at λ = 517 nm using a Hitachi UV 2000 spectrophotometer (Hitachi, Tokyo, Japan). Results were expressed as the percentage of DPPH^•^ bleaching, and reported as IC_50_ values (mean ± S.D.) from three independent experiments.

### 2.6. SOD-like Activity Assay

The superoxide anion scavenging assay was conducted spectrophotometrically, as described by Di Giacomo et al. [30]. The assay relies on the superoxide anion in vitro production by a reaction mix composed of 100 mM triethanolamine–diethanolamine buffer (pH 7.4), 3 mM NADH, 25 mM EDTA/12.5 mM MnCl_2_, and 10 mM β-mercaptoethanol, in the presence or absence of different CCE concentrations. The reaction mix was incubated for 20 min, at room temperature, in the darkness, before measuring the decrease in NADH absorbance at λ = 340 nm using a Hitachi UV-2000 spectrophotometer (Hitachi, Tokyo, Japan). Results were calculated as the percentage inhibition of NADH oxidation and reported as IC_50_ values (mean ± S.D.) from three independent experiments.

### 2.7. Catalase-like Activity Assay

The hydrogen peroxide scavenging assay was evaluated as described by Bianchi et al. [31]. Briefly, different CCE concentrations were mixed with 24 mM H_2_O_2_ in a final volume of 1 mL. Phosphate buffer (pH 7.4) was used as a solvent for the reaction mix, which was incubated for 10 min, at room temperature, in the dark, before measuring the absorbance at λ = 240 nm using a Hitachi UV 2000 spectrophotometer (Hitachi, Tokyo, Japan). Results were expressed as the percentage reduction in absorbance relative to the control and reported as IC_50_ values (mean ± S.D.) from three independent experiments.

### 2.8. Cell Cultures and Treatments

CaCo-2 cells (human colon adenocarcinoma, ATCC HTB-37^TM^, Manassas, VA, USA) were cultured in Minimum Essential Medium (MEM, Gibco 11095-080, Thermo Fisher Scienfitic, Milan, Italy) supplemented with 1 mM pyruvate, 2 mM L-glutamine, 100 U/mL penicillin, 100 μg/mL streptomycin, 1% non-essential amino acids, and 10% fetal bovine serum (FBS, Gibco A5256701, Thermo Fisher Scienfitic, Milan, Italy). HFF-1 cells (human fibroblasts, ATCC SCRC-1041^TM^, Manassas, VA, USA) were maintained in high-glucose Dulbecco’s Modified Eagle Medium (DMEM, 4.5 g/L, Gibco 41966-029, Thermo Fisher Scienfitic, Milan, Italy) supplemented with 1 mM pyruvate, 4 mM L-glutamine, 100 U/mL penicillin, 100 μg/mL streptomycin, and 10% FBS. Cells were incubated at 37 °C in a humidified atmosphere with 5% CO_2_. Cells were harvested when approximately 80% confluence was reached using 0.25% trypsin–EDTA solution (Gibco 25200-056, Thermo Fisher Scienfitic, Milan, Italy). Trypsin was inactivated by adding three volumes of complete medium and a cell pellet was obtained by centrifuging at 300× *g* for 5 min. The cell pellet was then resuspended in fresh complete medium and either expanded in flasks or seeded for subsequent experiments. CCE stock solution was freshly prepared at 1 mg/mL in cell culture medium immediately prior to use. The solution was then diluted in cell culture medium to obtain the final concentrations tested (5, 7.5, 10, 12.5, 15, 17.5, 20, 50, and 100 μg/mL). Similarly, 5-FU stock solution was prepared in cell culture medium at 1 mg/mL and stored at −20 °C for up to one month, with freeze–thaw cycles avoided. Prior to use, the 5-FU stock solution was diluted in cell culture medium to achieve the final concentrations tested (0.1, 0.5, 1, and 10 μg/mL).

### 2.9. MTT Assay

Cell viability was evaluated using the MTT assay, which quantifies the conversion of tetrazolium salt to formazan in metabolically active cells. The amount of formazan produced is directly proportional to the number of viable cells. Absorbance of the converted formazan was measured with a microplate spectrophotometer reader (Titertek Multiskan, Flow Laboratories, Helsinki, Finland) at 570 nm [31]. CaCo-2 cells were seeded in 96-well plates at 1 × 10^4^ cells per well and, after 24 h, treated with increasing concentrations of CCE (5, 7.5, 10, 12.5, 15, 17.5, and 20 μg/mL) for 24 h. Additionally, CaCo-2 cells were treated with 5-FU (0.1, 0.5, 1 and 10 μg/mL) alone or in combination with CCE (5, 7.5, 10, and 12.5 μg/mL) for 72 h. HFF-1 cells, used as a control cell line, were seeded in 96-well plates at 7 × 10^3^ cells per well and, after 24 h, treated with increasing concentrations of CCE (50 and 100 μg/mL) for 72 h. Results are expressed as % of cell viability vs. untreated control cells and presented as the mean ± S.D. of four independent experiments.

### 2.10. Lactic Dehydrogenase Release

CaCo-2 cells were seeded in 24-well plates at 3 × 10^5^ cells per well and, after 24 h, were treated with two concentrations (7.5 and 12.5 μg/mL) of CCE. Following 24 h of treatment, lactate dehydrogenase (LDH) release was evaluated as an indicator of cell necrosis due to membrane damage. Enzymatic activity was quantified spectrophotometrically in both culture medium and cell lysates by measuring NADH reduction at λ = 340 nm [32]. The proportion of LDH released was calculated as the ratio of the total enzymatic activity to that in the corresponding culture medium. Data are reported as the percentage of total LDH released ± S.D. of three independent experiments.

### 2.11. Reactive Oxygen Species Assay

CaCo-2 cells were seeded in 24-well plates at 3 × 10^5^ cells per well. After 24 h, cells were treated with two concentrations (10 and 12.5 μg/mL) of CCE and intracellular ROS levels were quantified after 24 h of treatment, using a modified H_2_DCFDA assay as described by Malfa et al. [33]. Cells were incubated with the H_2_DCF-DA probe (5 μM) for 30 min at 37 °C and then washed with ice-cold Ca^2+^/Mg^2+^-free PBS, two times, and lysed by incubating for 1h at 4 °C with 250 μL per well of digitonin solution (2.5 mg/mL). Lysates were clarified by centrifugation at 12,000× *g* for 10 min at 4 °C and the intensity of fluorescence (I.F.) was measured on 100 μL of the supernatant using a Sinergy HT Biotek (Agilent Technologies, Milan, Italy) microplate reader (λ_ex_ = 488 nm, λ_em_ = 525 nm). Fluorescence measurements were normalized to total protein content and expressed as a percentage of untreated controls. Experiments were performed in quadruplicate, and the results are presented as the mean ± S.D.

### 2.12. Total Thiol Group Determination

CaCo-2 cells were seeded in 24-well plates at 3 × 10^5^ cells per well. After 24 h, cells were treated with two concentrations (10 and 12.5 μg/mL) of CCE and non-protein total thiol groups (RSH) were quantified after a 24 h treatment using a spectrophotometric assay based on the reaction of thiol groups with 2,2-dithio-bisnitrobenzoic acid (DTNB) at 412 nm [33]. To perform the experiment, cells were harvested and lysed by sonication. Then, 200 μL of cell lysate was added to a reaction mix consisting of 600 μL of TRIS base (0.25 M, pH 8.2), and 400 μL of DTNB (10 mM) absolute ethanol solution. The reaction mix was incubated for 20 min at room temperature and centrifuged at 3000× *g* for 10 min. The absorbance of the supernatant was measured using a Hitachi UV 2000 spectrophotometer (Hitachi, Tokyo, Japan) and compared to a GSH calibration curve to determine the total thiol concentration. Results are expressed as nmol of RSH/mg of protein ±S.D. of four independent experiments.

### 2.13. Western Blotting Analysis

Lysates of CaCo-2 cells (3 × 10^5^ cells/mL) were prepared using radioimmunoprecipitation assay (RIPA) buffer containing a phosphatase and protease inhibitor cocktail. Protein content was quantified using the Bradford method. Western blot analysis was performed as previously described [34] with the following primary antibodies: anti-Nrf2 (PA5-88084, Thermo Fisher Scientific, Milan, Italy 1:1000 dilution), anti-p53 (ab26, Abcam, Cambridge, UK, 1:1000 dilution), anti-Bax (sc493, Santa Cruz Biotechnology, Dallas, TX, USA, 1:300 dilution), anti-Cytochrome C (Cat #11940, Cell Signaling Technology, Danvers, MA, USA, 1:800 dilution), anti-Caspase 3 (ab32351, Abcam, Cambridge, UK, 1:700 dilution), and anti-βActin (ab 179467, Abcam, Cambridge, UK, 1:1000 dilution). Secondary antibodies included goat anti-rabbit labeled with IRDye 680 (1:15,000) and goat anti-mouse labeled with IRDye 800 (1:20,000) for detection. The Odyssey Infrared Imaging System (LI-COR Biosciences, Lincoln, NE, USA) was used to scan the blot, and quantitative densitometric analysis was performed by using ImageJ software (version 1.53g; National Institutes of Health, Bethesda, MD, USA). The results are expressed as arbitrary densitometric units (A.D.U.) and normalized to actin levels. Western blot original images are reported in Supplementary Materials.

### 2.14. Combination Index Analysis and Normalized Isobologram

The combination Index (CI) and normalized isobologram for non-constant ratio combinations were calculated as reported by Espana-Serrano and Chougule [35]. CI values at 50% of efficacy were calculated by adding the concentrations, expressed as IC_50_ eq., which resulted in 50% of cell viability reduction in CaCo-2 when used in combination. 5-FU IC_50_ eq. were calculated by dividing the concentrations used in combination (0.1–0.5–1 μg/mL) by the IC_50_ for the drug alone (5.46 μg/mL). CCE IC_50_ eq. were calculated by dividing the IC_50_ obtained when in combination with the previously reported fixed 5-FU concentration (respectively, 7.63–5.88–4.84 μg/mL) by the IC_50_ for the extract alone (9.31 μg/mL). CI values are interpreted as: CI > 1.1 antagonism; CI = 0.9–1.1 additive; CI = 0.8–0.9 slight synergism; CI = 0.6–0.8 moderate synergism; CI = 0.4–0.6 synergism; CI = 0.2–0.4 strong synergism. The normalized isobologram was created by plotting points on a cartesian plane with coordinates (x; y), where the x-axis represents the 5-FU concentrations and the y-axis the CCE concentrations, both expressed as IC_50_ eq. The additivity line was drawn by connecting the points representing the IC_50_ values of the single treatments expressed as IC_50_ eq. with coordinates (0; 1) for 5-FU and (1; 0) for CCE. The concentrations resulting in 50% of cell viability were plotted, with coordinates (5-FU IC_50_ eq.; CCE IC_50_ eq.), calculated as described above. Depending on the position of the points, the graph was graphically interpreted as above the line: antagonistic interaction; on the line: additive interaction; below the line: synergistic interaction.

### 2.15. In Vitro Antimicrobial Activity Against Pathogens

The ability of CCE to exert antimicrobial activity was evaluated by standard agar disc diffusion assay following the protocol reported by Cioni and co-workers with slight modifications [36]. *Escherichia coli* ATCC 10536, *Escherichia coli* ATCC 25922, *Enterobacter cloacae* DMS 30054, *Staphylococcus aureus* ATCC 6538, *Enterococcus faecalis* ATCC 29212, and *Pseudomonas aeruginosa* DSM 1117 were used as target opportunistic pathogens. Each target strain was cultured using the media and following the conditions suggested by the ATCC or DSM. In detail, pure colonies of each target microorganism were suspended in sterile saline solution to reach a turbidity matching the McFarland 0.5. Each standardized organism was swabbed onto Muller Hinton agar (Oxoid, Milan, Italy) and sterile paper discs (6 mm in diameter), impregnated with 100 μL of CCE at a concentration of 10.0 mg/mL, were placed on the surface of the inoculated plates. After incubation for 24 h at 37 °C, the plates were examined for the presence of inhibition halos around the cardboard discs. The antibiotics vancomycin (30 μg/disc) and ciprofloxacin (5 μg/disc) were used as positive controls to validate assay performance and verify strain susceptibility. Triplicate experiments were performed, and the antagonistic activity was evaluated by measuring the zones of inhibition in mm, excluding the diameter of the paper disc. The antimicrobial activity was ranked as absent (no inhibition zone); low (inhibition zone < 10 mm); intermediate (inhibition zone between 11 and 20 mm); and high (inhibition zone > 20 mm) and the results are reported as the mean ± SD of three experiments.

### 2.16. Statistical Analysis

Statistical analyses were performed by one-way ANOVA coupled with Tukey’s multiple comparison test. *p* < 0.05 was considered significant.

## 3. Results

### 3.1. Phytochemical and Antioxidant Characterization of CCE

#### 3.1.1. Phytochemical Analysis

Phytochemical characterization of CCE was conducted to determine its bioactive compound profile. Both quantitative and qualitative analyses were performed using spectrophotometry and high-performance liquid chromatography with a diode-array detector (HPLC-DAD).

Spectrophotometric analysis revealed that the TPC and TFC of CCE were 178.33 ± 2.06 mg gallic acid eq. (GAE) per gram of extract and 52.21 ± 1.48 mg catechin eq. (CE) per gram of extract, respectively.

The phytochemical profile of CCE was analyzed by HPLC-DAD, allowing characterization of the principal secondary metabolites. The chromatogram indicated that phenolic acids, especially caffeoylquinic acid derivatives, were predominant, accompanied by various flavonoid compounds.

**Figure 2:**
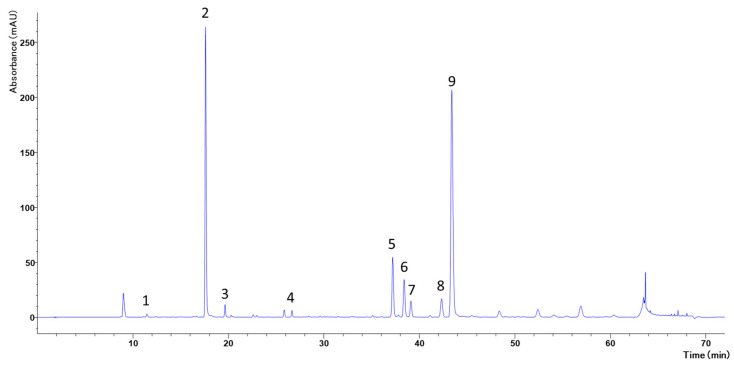
Phytochemical profiles of polyphenols by HPLC-DAD of CCE.

Several luteolin derivatives and apigenin were identified among these compounds, both of which are recognized for their biological activities. Figure 2 presents the distribution and intensity of the corresponding peaks, and Table 1 lists the identified metabolites.

#### 3.1.2. In Vitro Cell-Free Antioxidant Properties

In addition to phytochemical characterization, the in vitro antioxidant activity of CCE was systematically evaluated using several complementary assays to ensure a comprehensive assessment of its redox potential. The assays employed were the DPPH assay, which measures the scavenging capacity for stable free radicals, the assessment of superoxide dismutase-like (SOD-like) activity, which reflects the ability to dismutate superoxide anions, and the analysis of catalase-like activity, which quantifies the extract efficiency in decomposing hydrogen peroxide. As shown in Table 2, CCE exhibits good antioxidant activity across all tested systems. The IC_50_ values found are, respectively, for the DPPH test 21.35 ± 1.92 µg/mL, SOD-like 1.56 ± 0.4 µg/mL, and catalase-like 314.73 ± 2.26 µg/mL. These findings demonstrate a strong capacity to neutralize various reactive species and suggest a synergistic effect arising from the phenolic and flavonoid compounds present in the extract matrix.

### 3.2. Cell Viability

#### 3.2.1. CCE Cytotoxicity in CaCo-2 Cells

**Figure 3:**
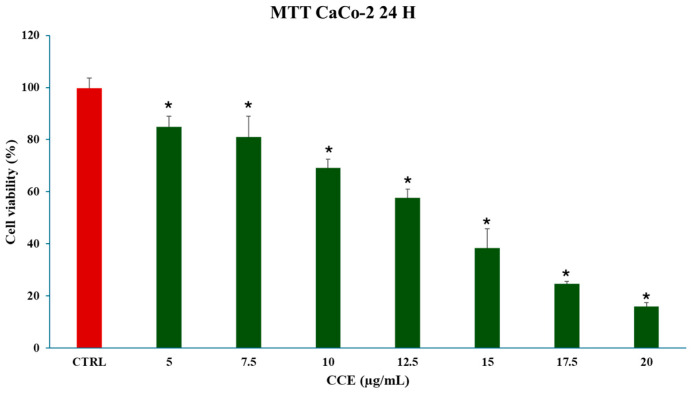
Effect of CCE on CaCo-2 cell viability after a 24 h treatment with different extract concentrations (from 5 to 20 μg/mL). Data are represented as the means ± S.D. of four independent experiments. Confidence intervals were calculated by one-way ANOVA test: * Significant vs. untreated control cells; *p* < 0.05.

The effect of CCE was assessed in CaCo-2 cells using an MTT assay after exposure to different extract concentrations (5, 7.5, 10, 12.5, 15, 17.5, 20 μg/mL) for 24 h. The results, presented in Figure 3, show that the extract exerted potent, concentration-dependent cytotoxicity on this cancer cell line, significantly reducing cellular viability at 5 μg/mL and achieving an IC_50_ of 13.07 ± 0.64 μg/mL.

#### 3.2.2. CCE Cytotoxicity in HFF-1 Cells

**Figure 4:**
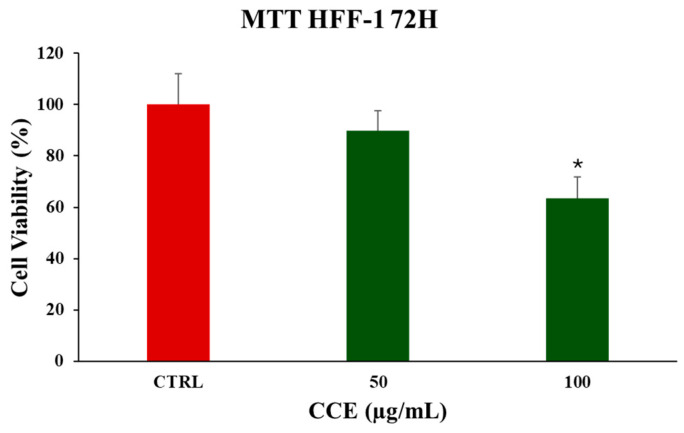
Effect of CCE on HFF-1 cell viability after a 72 h treatment with different extract concentrations (50–100 μg/mL). Data are represented as the means ± S.D. of four independent experiments. Confidence intervals were calculated by one-way ANOVA test: * Significant vs. untreated control cells; *p* < 0.05.

The safety profile of the extract was evaluated using HFF-1, a non-cancerous human fibroblast cell line. Significant reduction in cell viability was observed only at 100 μg/mL following 72 h of treatment (Figure 4). These results indicate that the extract possesses minimal cytotoxicity toward normal cells, underscoring its potential selectivity for cancerous cells.

### 3.3. Redox Homeostasis

#### 3.3.1. Effect of CCE on ROS and RSH Levels in CaCo-2 Cells

**Figure 5:**
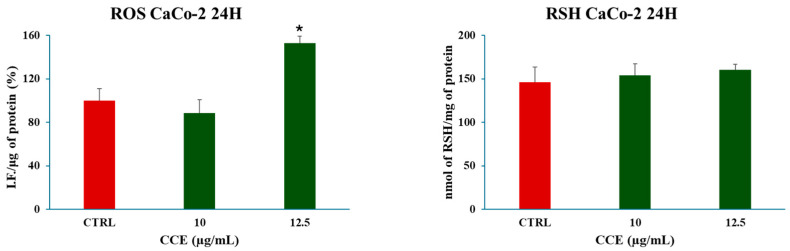
Effect of CCE extract on ROS and RSH levels after a 24 h treatment with different extract concentrations (10–12.5 μg/mL). Data are represented as the means ± S.D. of four independent experiments. Confidence intervals were calculated by one-way ANOVA test: * Significant vs. untreated control cells; *p* < 0.05.

As a possible mechanism of action for the induction of cell death, the effect of CCE on cancer cell redox homeostasis was assessed by measuring the main cellular oxidant species (ROS) and non-enzymatic antioxidant defenses (GSH). The latter was evaluated by measuring the total non-protein thiol groups (RSH), of which GSH is the most abundant [37]. Concentrations below the IC_50_ which caused a significant decrease in cell viability (10–12.5 μg/mL) were chosen to perform the experiments. No differences were found between control and treated cells after 24 h for RSH, while a significative increase was detected in the level of ROS with the concentration of 12.5 μg/mL (Figure 5).

#### 3.3.2. Effect of CCE on Nrf-2 Expression in CaCo-2 Cells

**Figure 6:**
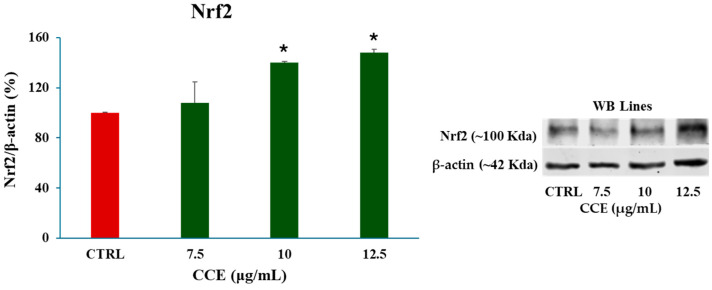
Effect of CCE on Nrf-2 expression after a 48 h treatment with different extract concentrations (7.5–10–12.5 μg/mL). Data are represented as the means ± S.D. of three independent experiments. Confidence intervals were calculated by one-way ANOVA test: * Significant vs. untreated control cells; *p* < 0.05.

These results are corroborated by the expression levels of Nrf-2, which increased its expression levels after 48 h of treatment with CCE (7.5–10–12.5 μg/mL) (Figure 6). This finding suggests that, under the tested conditions, the extract significantly modulates ROS levels, inducing the activation of the Nrf-2-mediated antioxidant response pathway.

### 3.4. Cell Death

#### 3.4.1. LDH Release

**Figure 7:**
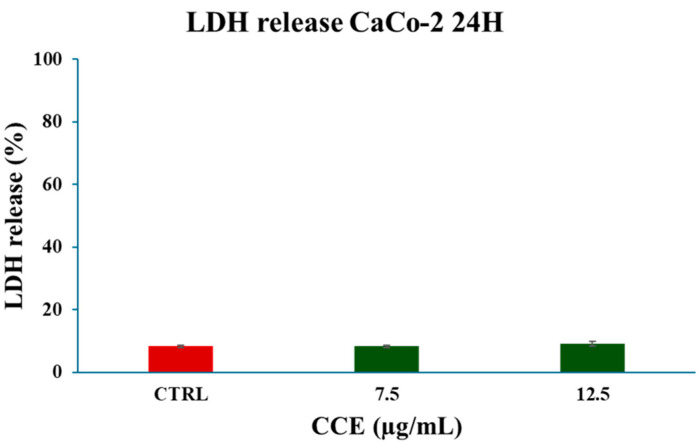
Effect of CCE on LDH release after a 24 h treatment with different extract concentrations (7.5–12.5 μg/mL). Data are represented as the means ± S.D. of three independent experiments. Confidence intervals were calculated by one-way ANOVA test.

The mechanism of cell death was firstly investigated by evaluating the release of LDH in culture medium, which is a marker of cell membrane disruption. Concentrations below the IC_50_ (7.5–12.5 μg/mL) were chosen to perform the experiment. The results, showed in Figure 7, showed no significant increase in LDH release following a 24 h treatment with CCE, allowing us to exclude necrosis and necrosis-like cell death mechanisms.

#### 3.4.2. Expression Levels of p53 and Bax

**Figure 8:**
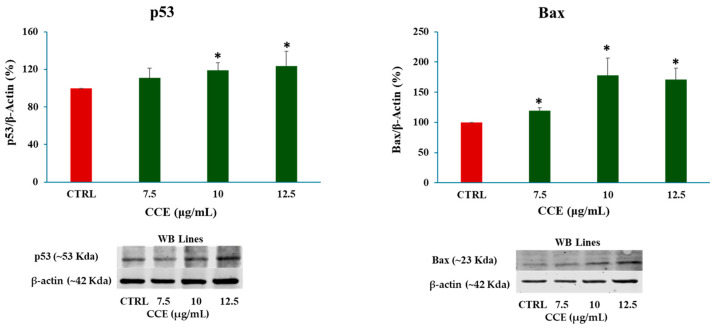
Effect of CCE on p53 and Bax expression after a 48 h treatment with different extract concentrations (7.5–10–12.5 μg/mL). Data are represented as the means ± S.D. of three independent experiments. Confidence intervals were calculated by one-way ANOVA test: * Significant vs. untreated control cells; *p* < 0.05.

Once necrosis was excluded, apoptotic cell death was investigated by measuring the expression of p53 and Bax by Western blot analysis after 48 h of treatment with CCE concentrations below the IC_50_ (7.5–10–12.5 μg/mL). As presented in Figure 8, both markers were upregulated in a concentration-dependent manner, suggesting that an apoptotic cell death program was initiated in treated CaCo-2 cells.

#### 3.4.3. Expression Levels of Cytochrome C and Caspase 3

**Figure 9:**
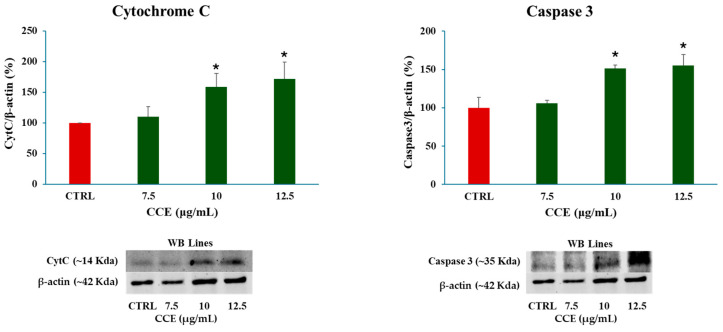
Effect of CCE on mitochondrial cytochrome c release and caspase 3 expression after 48 h treatment with different extract concentrations (7.5–10–12.5 μg/mL). Data are presented as the means ± S.D. of three independent experiments. Confidence intervals were calculated by one-way ANOVA test: * Significant vs. untreated control cells; *p* < 0.05.

The activation of the intrinsic apoptotic pathway was further confirmed by assessing mitochondrial cytochrome c release and caspase-3 expression. As shown in Figure 9, the results revealed a significant increase in both apoptotic markers, consistent with mitochondria-mediated apoptosis induction.

### 3.5. Chemosensitizing Effect

#### 3.5.1. Combination Cytotoxicity of CCE and 5-FU on CaCo-2 Cells

**Figure 10:**
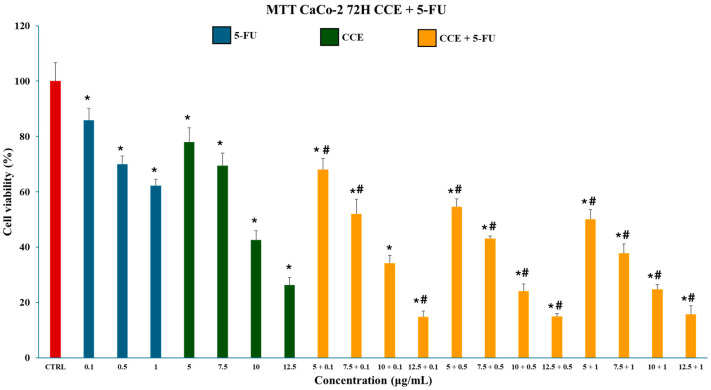
Effect of CCE (5–7.5–10–12.5 μg/mL), 5-FU (0.1–0.5–1 μg/mL), and combo treatments on CaCo-2 cell viability after a 72 h treatment. Data are represented as the means ± S.D. of four independent experiments. Confidence intervals were calculated by one-way ANOVA test: * Significant vs. untreated control cells; # significant vs. single treatments at the same concentration; *p* < 0.05.

The possible capability of CCE to enhance the cytotoxic activity of 5-FU was explored by cotreating CaCo-2 cells with a combination of different extract (5–7.5–10–12.5 μg/mL) and 5-FU (0.1–0.5–1 μg/mL) concentrations for 72 h. Chemotherapeutic concentrations below the IC_50_ (5.46 ± 0.76 μg/mL) were selected along with a treatment duration of 72 h as this is the time necessary for 5-FU to significantly exert its cytotoxic activity. Two concentrations below (5–7.5 μg/mL) and two above (10–12.5 μg/mL) the CCE IC_50_ at 72 h (9.31 ± 0.27 μg/mL) were selected for the combination. The results, presented in Figure 10, demonstrated that all the concentration combos, except for 10 + 0.1 μg/mL (CCE+5-FU), were able to significantly reduce the cell viability with respect to the single treatments.

#### 3.5.2. Synergistic Interaction of CCE and 5-FU: CI and Isobologram Analysis

**Figure 11:**
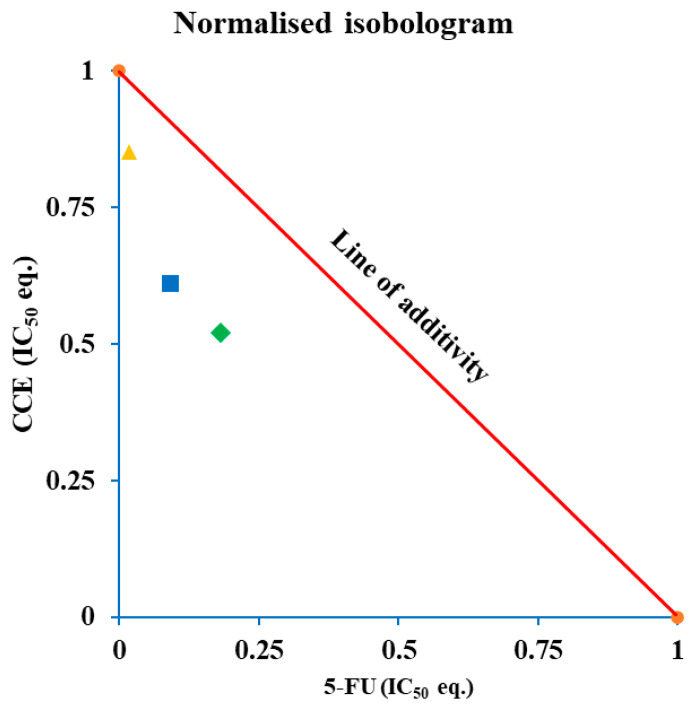
Normalized isobologram for nonconstant combination of 5-FU and CCE. Each point has coordinates (x; y), where y is the concentration of CCE, expressed as IC_50_ eq., which resulted in 50% of cell viability when combined with different 5-FU concentrations x of 0.018 (▲), 0.09 (■), and 0.18 (◆) IC_50_ eq., corresponding, respectively, to 0.1, 0.5, and 1 μg/mL. Data are calculated as the mean of four independent experiments. Depending on their position with respect to the line, drawn by connecting the normalized IC_50_ values of the two single treatments, points are interpreted as: above the line, antagonistic interaction; on the line, additive interaction; below the line, synergistic interaction.

Whether the effect was synergistic, additive or antagonistic, it was explored by calculating CI and generating normalized isobolograms for nonconstant ratio combinations. In the isobologram plot (Figure 11), all the points are below the line of additivity, suggesting a synergistic mechanism between the two components. This is further evidenced by the CI values, reported in Table 3, all less than 1 (0.87–0.70–0.70), resulting in a slight–moderate synergistic activity.

### 3.6. Antagonistic Activity Against Pathogens

The results of the antimicrobial activity exhibited by CCE (10.0 mg/mL) against the target opportunistic pathogens are reported in Table 4. The antimicrobial activity was ranked as absent (no inhibition zone); low (inhibition zone < 10 mm); intermediate (inhibition zone between 11 and 20 mm); and high (inhibition zone > 20 mm). Overall, detectable antimicrobial activity against both Gram-positive and Gram-negative strains was observed. Intermediate antimicrobial activity, with an inhibition zone between 11 and 20 mm, was recorded against all the tested pathogens with the exception of *Escherichia coli* ATCC 10536, showing low susceptibility (9.0 ± 1.63 mm). The inhibition zones obtained with antibiotics used as positive controls (ciprofloxacin, 5 µg/disc; vancomycin, 30 µg/disc) were consistent with expected susceptibility profiles, confirming assay reliability and strain responsiveness under the adopted experimental conditions.

## 4. Discussion

Natural compounds are emerging as promising agents for cancer prevention and treatment [38]. Specifically, several scientific papers are exploring and demonstrating the role of polyphenols in limiting the onset, progression, and invasiveness of CRC, as well as in acting as chemosensitizing agents [39,40]. For this reason, we decided to explore the possible cytotoxic activity of a hydroalcoholic extract prepared from *C. cardunculus* subsp. *cardunculus* aerial parts on the CRC model cell line CaCo-2. Initially, we phytochemically characterized CCE by spectrophotometric and chromatographic techniques. Specifically, colorimetric tests revealed a remarkable amount of total polyphenols (178.33 mg GAE/g) and flavonoids (52.21 mg CE/g), while the HPLC-DAD analysis displayed a rich phenolic profile comprising nine compounds, with chlorogenic acid as the most abundant, followed by dicaffeoylquinic acids, luteolin, and apigenin derivatives. Coherently, this phytocomplex is in line with the existing scientific literature on the genus *Cynara* [41] and with results we obtained in a previous study on *C. cardunculus* leaf aqueous extract [28]. All together, these results further confirmed wild artichoke’s richness in phenolic compounds compared to cultivated varieties [28].

We also characterized CCE from an antioxidant point of view, by testing its activity on three reactive species: DPPH^•^, ^−•^O_2_ (superoxide anion), and H_2_O_2_ (hydrogen peroxide). DPPH^•^ is a synthetic radical commonly used to screen the scavenging activity of natural compounds [42,43], while ^−•^O_2_ and H_2_O_2_ are two physiological ROS, involved in the cellular production of ^•^OH (hydroxyl radical) by the Haber–Weiss reaction catalyzed by transition metals [44]. The results showed potent activity of CCE against DPPH^•^ (IC_50_ 21.35 µg/mL) and ^−•^O_2_ (IC_50_ 1.56 µg/mL), while a moderate effect was observed on its capacity to decompose H_2_O_2_ (IC_50_ 314.73 µg/mL). Overall, these results suggest that the CCE extract exhibits good antioxidant activity, mainly through free radical scavenging.

**Figure 12:**
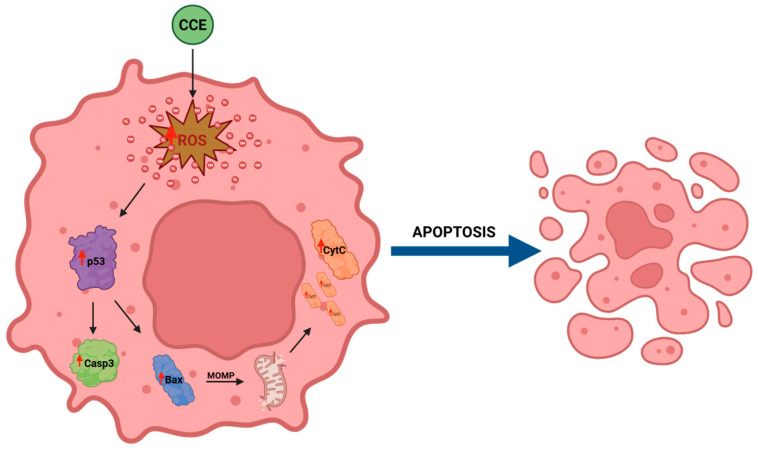
Proposed pathway involved in CCE mechanism of action. Created in BioRender. Bianchi, S. (2026) https://BioRender.com/z71lp1t. Accessed on 20 February 2026.

We began evaluating the cytotoxicity of CCE on the CRC cell line CaCo-2 by MTT assay. The results already showed potent activity after 24 h of treatment, with an IC_50_ of 13.07 ± 0.64 μg/mL. The safety of the extract was also assessed by treating non-cancerous human fibroblasts HFF-1, commonly used as a normal control cell line [45,46], with CCE. The extract showed remarkable selectivity towards cancer cells, resulting in moderate toxicity only after 72 h at the highest concentration tested (100 μg/mL). It is well known that polyphenols can exert dual action as anti- or prooxidants, depending on the redox microenvironment [47]. Specifically, in cancer cells, polyphenols have been often observed to increase intracellular ROS production and induce oxidative stress-triggered cell death [48]. Therefore, we explored the hypothesis of whether the activity of CCE was linked to a dysregulation of redox homeostasis in CaCo-2 cells by measuring the levels of one of the main classes of reactive species and the major non-enzymatic endogenous antioxidants in cells, namely, ROS and GSH [37,44]. Our results showed no change in GSH, but a significant increase in ROS after 24 h of treatment. These results suggest that CCE-induced cell death was driven by an alteration in cancer cell redox homeostasis, characterized by increased ROS but without changes in GSH levels. This effect is coherent with the CCE phytochemical profile, as chlorogenic acid and luteolin 7-glucoside have been found to increase ROS levels in cancer cells, triggering apoptotic cell death [49,50]. Consequently, CCE triggered the Nrf2-mediated antioxidant response pathway, as evidenced by its increased expression. This redox perturbation can also partially explain the safety of CCE, as it is known that polyphenol-induced oxidative cell death is selective for cancer cells [47]. We further investigated the mechanism of cell death, measuring the release of LDH in culture medium consequent to the treatment, which is a marker of cell membrane disruption, an event typical of necrosis and necrosis-like cell death, such as necroptosis and pyroptosis [51,52,53]. The findings allowed these mechanisms to be excluded, as no difference was found between the control group and the treated cells. Therefore, we evaluated apoptosis as a possible mechanism, considering that it is one of the main types of cell death induced by polyphenols in cancer cells [54,55], by evaluating the expression of two pro-apoptotic proteins (p53 and Bax), the mitochondrial release of Cytochrome C, and the increasing expression of Caspase-3 [56,57,58]. p53 is a transcription factor that regulates the expression of a wide range of genes, including those involved in autophagy, cell cycle arrest, and apoptosis. Therefore, p53 is an important regulator of cell fate under stress conditions, determining whether cells die or not. In normal conditions, p53 activity is suppressed by ubiquitination induced by MDM2 and MDMX, but in response to stressors this process is inhibited [59]. Polyphenols can upregulate p53, prevent its ubiquitination, and promote its transcriptional activity, increasing the expression of pro-apoptotic genes, including Bax, leading to apoptotic cell death [55,60,61]. Bax is a member of the Bcl-2 protein family, an effector of intrinsic apoptosis. Normally, Bax is inactivated by binding to anti-apoptotic Bcl-2 proteins; however, when the equilibrium within the Bcl-2 family is perturbed by a pro-apoptotic stressor, Bax can homo-oligomerize, causing pore formation and consequent mitochondrial outer membrane permeabilization (MOMP) [56]. Bax activity is linked with p53 not only because, as mentioned above, it can increase its expression, but also because p53 can bind anti-apoptotic Bcl-2 proteins, inhibiting their activity [56]. In this context, polyphenols have been shown to trigger apoptosis in cancer cells by increasing Bax expression [55,60]. Cytochrome c is a redox-active protein located in the mitochondrial intermembrane space that serves as an electron shuttle from complex III to complex IV of the mitochondrial respiratory chain, thus playing an essential role in cellular bioenergetics [62]. MOMP leads to the release of Cytochrome c and apoptosome formation following binding with protease-activating factor-1 (APAF-1) [56]. This event initiates the caspase cascade, culminating in the cleavage of pro-caspase 3 and the subsequent activation of the apoptotic executioner caspase-3 [63]. Our results demonstrated increases in both Bax and p53, cytochrome c release, and caspase-3. Therefore, we suggest that CCE induces a pro-apoptotic program in CaCo-2 cells, probably driven by an increase in p53, which, in turn, upregulates the effector of intrinsic apoptosis, Bax, with consequent cytochrome c release and pro-caspase 3 cleavage. The proposed pathway involved in CCE activity is summarized in Figure 12.

These data are consistent with the existing scientific literature on the anticancer activity of CCE constituents. Chlorogenic acid and Apigenin 7-O-Glucoside have been reported to induce apoptosis with an increase in Bax and p53 in colon cancer cells [64,65]. Similar effects have been observed with neochlorogenic acid on gastric cancer cells, both in vitro and in vivo [66]. Luteolin 7-glucoside increases Bax expression and induces apoptosis in CRC cells [67], and Cynarine was found to induce apoptosis in breast cancer cells [68].

Furthermore, we decided to test the potential chemosensitizing activity of CCE for 5-FU, which is currently one of the most widely used drugs for CRC therapy [69]. The rationale behind this supposition lies in the upregulation of p53. Indeed, it has been shown that p53 downregulation reduces the responsiveness of CRC cells to 5-FU, whereas increased p53 expression improves their sensitivity [70,71]. The results corroborated our hypothesis, showing a synergistic interaction between 5-FU and CCE, quantified as slight-to-moderate synergy. Considering that CCE is a crude extract, this finding highlights the CCE phenolic profile as a possible chemosensitizer agent for CRC treatment. Finally, the antimicrobial activity against opportunistic pathogens exhibited by the tested extract, in line with previously reported data, indicated that CCE contains biologically active compounds that inhibit bacterial growth in vitro [72,73,74]. It is well known that *C. cardunculus* spp. are rich in polyphenol compounds, such as chlorogenic acid, apigenin, and luteolin, which are able to exhibit notable antibacterial and antifungal activities [73,75,76,77,78]. In line with the redox-modulating and pro-apoptotic effects observed in CaCo-2 cells, emerging evidence suggests that polyphenols may exert anticancer activity not only through direct cytotoxic mechanisms but also via microbiota-mediated pathways. Beyond direct cytotoxic effects, several polyphenols have also been reported to modulate gut microbial composition and inhibit opportunistic pathogens, suggesting a potential dual relevance in CRC prevention and management [79,80]. Increasing evidence indicates that gut microbiota dysbiosis contributes to colorectal carcinogenesis by promoting chronic inflammation, oxidative stress, and tumor-promoting signaling pathways, while microbial composition affects the response to cancer therapy and intestinal homeostasis during treatment [81,82]. In this context, the antimicrobial activity exerted by CCE may represent an additional mechanism contributing to its anticancer potential. Consistent with the available literature, Gram-positive bacteria displayed higher susceptibility than Gram-negative species, likely due to differences in cell envelope structure and permeability that affect phenolic compound uptake, as well as the presence in the periplasmic space of enzymes that can break down molecules introduced from outside [72,73,74]. Although the disk diffusion assay provides initial screening, further work evaluating the minimum inhibitory concentration (MIC) and minimum bactericidal concentration (MBC) will be conducted to explore the extract’s antimicrobial spectrum. This evidence supports the potential use of CCE as a natural agent that can modulate opportunistic pathogens, particularly in the context of chemotherapy-induced gut dysbiosis. Therefore, the antimicrobial properties of CCE may complement its pro-apoptotic and chemosensitizing activities, supporting a multitarget mode of action relevant to CRC management. Further studies are ongoing to better understand the mechanism of action of the extract, alone and in combination with 5-FU and other CRC treatments, with the aim of showing its ability to augment the efficacy of the therapeutic approaches currently in use.

## 5. Conclusions

The hydroalcoholic leaf extract of *Cynara cardunculus* subsp. *cardunculus* showed high phenolic content and strong antioxidant activity in DPPH, SOD-like, and catalase-like tests. The CCE phytochemical characterization led to the identification of nine phenolic compounds, namely, neochlorogenic acid, chlorogenic acid, cryptochlorogenic acid, cynarine, luteolin 7-glucoside, luteolin 7-glucuronide, 3,4-dicaffeoylquinic acid, apigenin 7-O-Rutinoside, and 1,5-dicaffeoylquinic acid. These compounds are well known for their anticancer activities, therefore providing the rationale for what was explored in the present work. In CaCo-2 colorectal cancer cells, CCE caused cytotoxicity in a concentration-dependent manner (IC_50_ 13.07 μg/mL at 24 h). This effect was linked to higher levels of ROS, increased Nrf2, and apoptosis markers (p53, Bax, cytochrome c, and caspase-3). There was no sign of necrosis, since LDH release was not detected, and CCE was not toxic to normal HFF-1 fibroblasts at concentrations up to 50 μg/mL. CCE also worked moderately well with 5-fluorouracil (CI 0.70–0.87) and showed broad antimicrobial activity against enteric pathogens, especially Gram-positive strains.

These results show that CCE has selective antitumor effects by triggering mitochondria-driven apoptosis through oxidative stress and making cancer cells more sensitive to chemotherapy, which could allow for lower doses of 5-fluorouracil. Its antibacterial activity may also help to reduce chemotherapy-related gut problems. Even if the in vitro cancer model applied presents several limitations including the 2D geometry, and the fact that it overlooks drug absorption, distribution and metabolism and does not fully represent the tumor microenvironment, this study represents a valid first step for the evaluation of the phytocomplex from *C. cardunculus* as a promising chemosensitizing agent for colon cancer therapy. Moreover, the concentrations used in this study have physiological relevance. In fact, considering the average polyphenol absorption after oral intake [83], 900 mg of extract would be theoretically needed to reach a phenolic plasmatic concentration corresponding to the higher one tested for evaluating CCE’s mechanism of action. Additional in vivo studies and tests to find the minimum inhibitory and bactericidal concentrations are needed to better understand how CCE works and to assess its use in colorectal cancer treatment. This research supports the value of Mediterranean plant-based therapies.

## Figures and Tables

**Table 1:** Compounds identified in CCE by HPLC-DAD.

Peak	Compound	Wavelength (nm)	Ret. Time (min.)
1	Neochlorogenic acid	325	11.45
2	Chlorogenic acid	325	17.61
3	Cryptochlorogenic acid	325	19.64
4	Cynarine	325	26.64
5	Luteolin 7-Glucoside	346	37.20
6	Luteolin 7-Glucuronide	346	38.40
7	3,4-Dicaffeoylquinic acid	325	39.11
8	Apigenin 7-O-Rutinoside	325	42.33
9	1,5-Dicaffeoylquinic acid	346	43.45

**Table 2:** Total polyphenols, total flavonoids and IC_50_ values (μg/mL) of CCE on the three reactive species.

	TPC (mg GAE/g Extract)	TFC (mg CE/g Extract)	DPPH Test IC_50_ (µg/mL)	SOD-like IC_50_ (µg/mL)	Catalase-like IC_50_ (µg/mL)
CCE	178.33 ± 2.06	52.21 ± 1.48	21.35 ± 1.92	1.56 ± 0.4	314.73 ± 2.26

**Table 3:** Concentration of 5-FU and CCE in combination which resulted in 50% of cell viability and related combination index (CI). Data are calculated as the mean of four independent experiments.

Concentrations for 50% Cell Viability		Concentrations as IC_50_ eq.		CI *	Interpretation
5-FU	CCE	5-FU	CCE		
0.1	7.63 ± 0.44	0.018	0.85	0.87	slight synergism
0.5	5.88 ± 0.30	0.09	0.61	0.70	moderate synergism
1	4.84 ± 0.80	0.18	0.52	0.70	moderate synergism

* CI values are interpreted as: CI > 1.1 antagonism; CI = 0.9–1.1 additive; CI = 0.8–0.9 slight synergism; CI = 0.6–0.8 moderate synergism; CI = 0.4–0.6 synergism; CI = 0.2–0.4 strong synergism.

**Table 4:** Antimicrobial activity against selected pathogens exhibited by CCE.

Tested Pathogens	Inhibition Zone (mm)
*Enterococcus faecalis* ATCC 29212	17.66 ± 0.47
*Enterobacter cloacae* DMS 30054	11.40 ± 1.25
*Escherichia coli* ATCC 25922	11.33 ± 1.25
*Escherichia coli* ATCC 10536	9.0 ± 1.63
*Pseudomonas aeruginosa* DSM 1117	13.33 ± 1.25
*Staphylococcus aureus* ATCC 6538	17.0 ± 0.82

## Data Availability

Data were generated at Department of Drug and Health Science, University of Catania. Data supporting the results of this study are available from the corresponding authors on request.

## Supplementary Materials

The following supporting information can be downloaded at: https://www.mdpi.com/article/10.3390/biology15060475/s1; Original western blot images and overlapping spectra for phytochemicals identification.

## Abbreviations

The following abbreviations are used in this manuscript:

CRC	Colorectal cancer
HPLC	High-performance liquid chromatography
DAD	Diode-array detection analysis
TPC	Total phenolic content
GAE	Gallic acid equivalent
TFC	Total flavonoid content
CE	Catechin equivalent
CaCo-2	Human colon adenocarcinoma cell line
CCE	*Cynara cardunculus* subsp. *cardunculus* extract
EQ	Equivalents
ROS	Reactive oxygen species
NRF2	Nuclear factor erythroid 2-related factor 2
LDH	Lactate dehydrogenase
HFF-1	Non-cancerous human fibroblast cell line
5FU	5-Fluorouracil
DMSO	Dimethyl sulfoxide
DCFH-DA	2′,7′-dichlorodihydrofluorescein diacetate
DPPH	2,2-diphenyl-1-picrylhydrazyl
S.D.	Standard deviation
SOD	Superoxide dismutase
NADH	Nicotinamide adenine dinucleotide reduced form
MEM	Minimum essential medium
DMEM	Dulbecco’s modified Eagle medium
FBS	Fetal bovine serum
PBS	Phosphate-buffered saline
DTNB	2,2-dithio-bisnitrobenzoic acid
RIPA	Radioimmunoprecipitation assay buffer
A.D.U.	Arbitrary densitometric units
CI	Combination index
RSH	Non-protein thiol groups
GSH	Glutathione reduced form
