# Supplementary material for: Cynara cardunculus subsp. cardunculus (Wild Artichoke) Extract: Antimicrobial Activity and Cytotoxicity, Apoptosis Induction, and Chemosensitization in Colon Cancer Cells
Source: Biology (Basel). 2026 Mar 15;15(6):475. doi: 10.3390/biology15060475 (PMC13023877; doi:10.3390/biology15060475)

Cynara cardunculus subsp. *cardunculus* (Wild Artichoke) Extract: Antimicrobial Activity and Cytotoxicity, Apoptosis Induction, and Chemosensitization in Colon Cancer Cells

Simone Bianchi [1,2,†], Rosaria Acquaviva [1,2,3,†], Claudia Di Giacomo [1,2,*], Barbara Tomasello [1,2], Francesco Pappalardo [1], Alessandra Pino [2,4], Irina Naletova [5], Donata Condorelli [1], Alfonsina La Mantia [1], Ignazio Barbagallo [6], Cinzia Randazzo[2,4], and Giuseppe Antonio Malfa [1,2,3,*]

The identification of nine phenolic compounds in CCE has been carried out by comparison of retention time and UV-Vis spectra with standard compounds from our internal library. Overlapping spectra supporting the identification are reported below.

1. Neochlorogenic acid

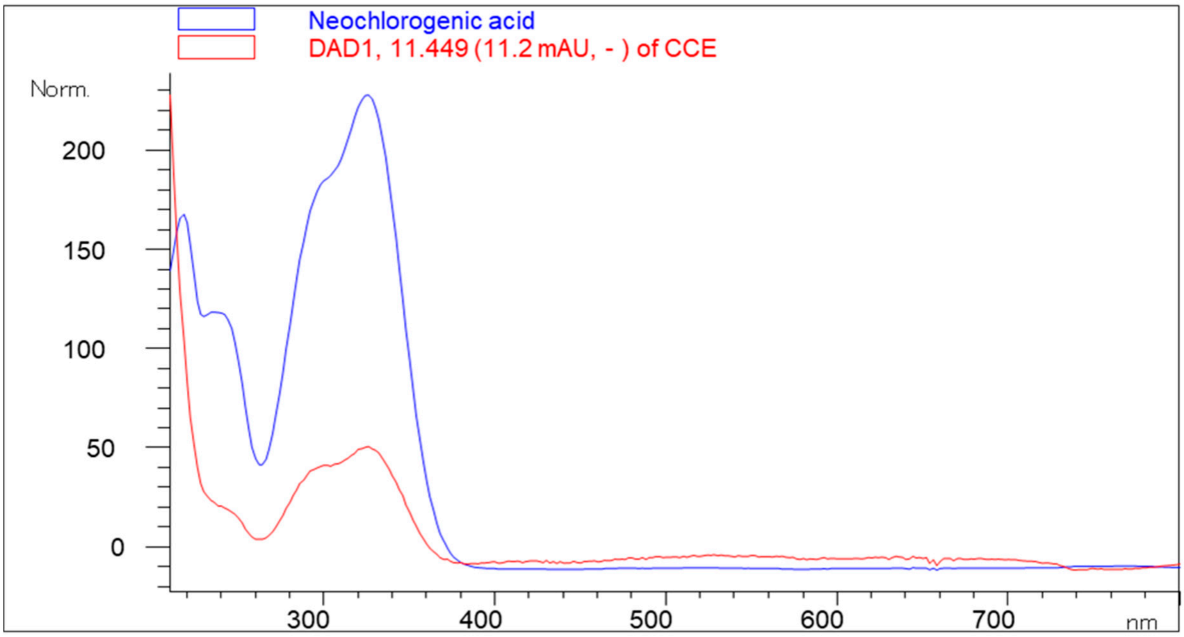

2. Chlorogenic acid

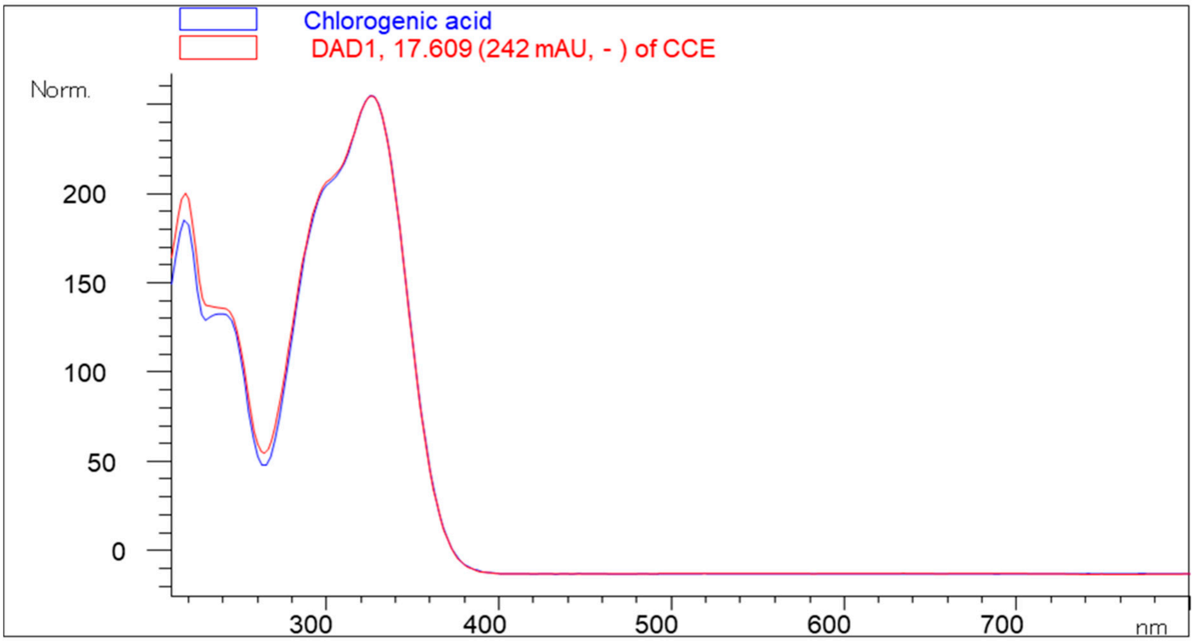

3. Cryptochlorogenic acid

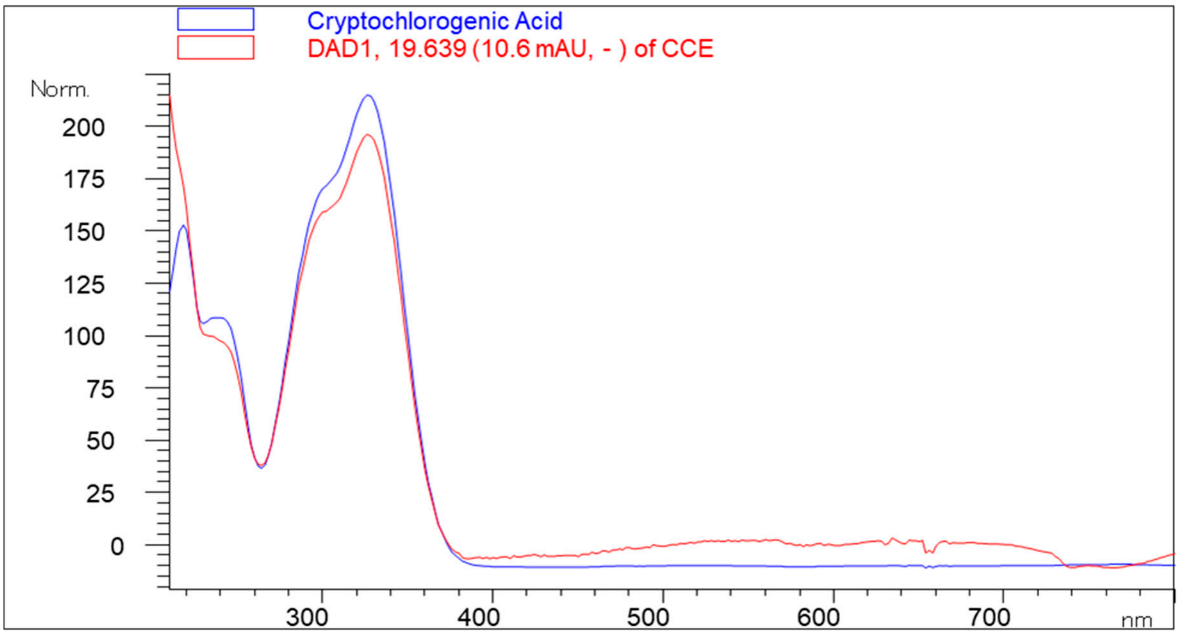

4. Cynarin

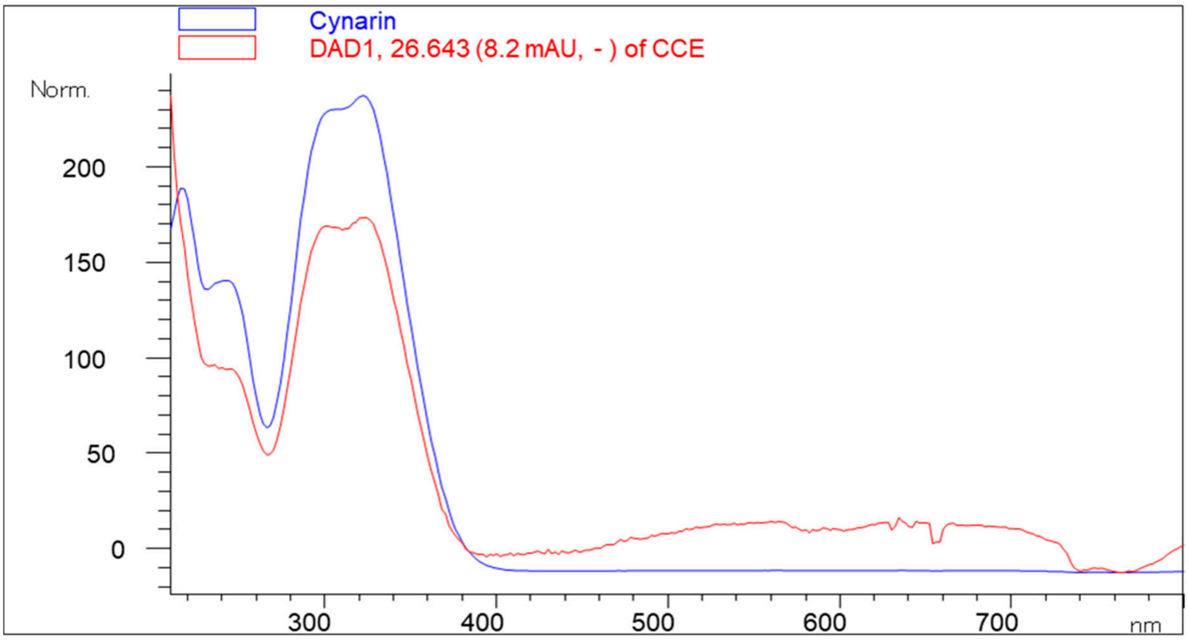

5. Luteolin 7-Glucoside

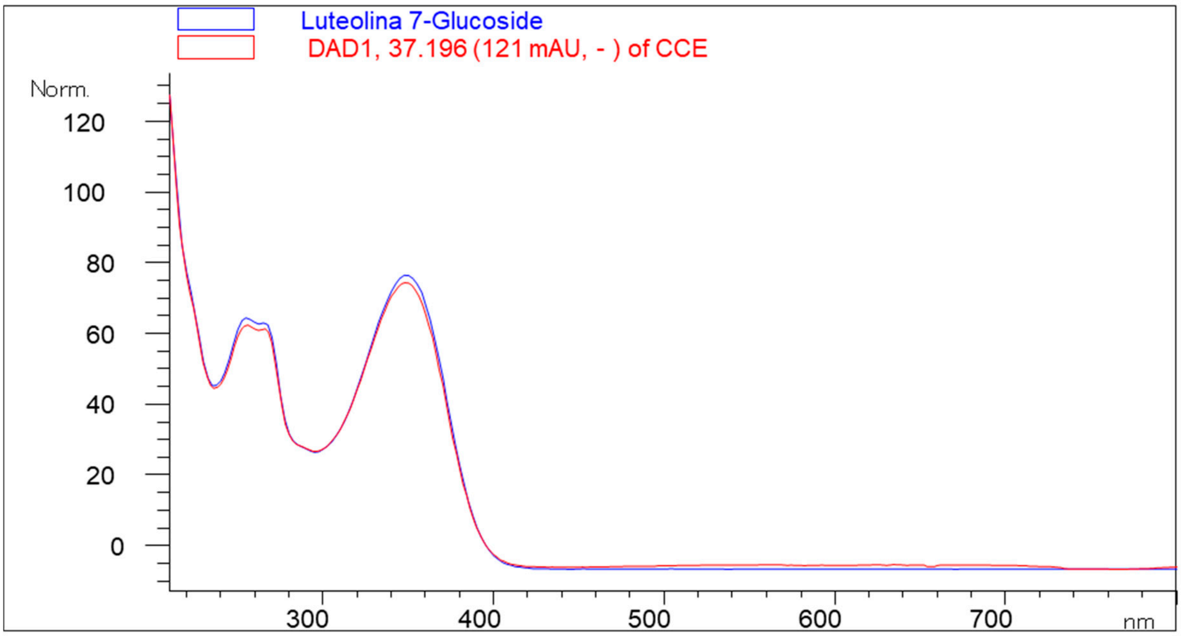

6. Luteolin 7-Glucuronide

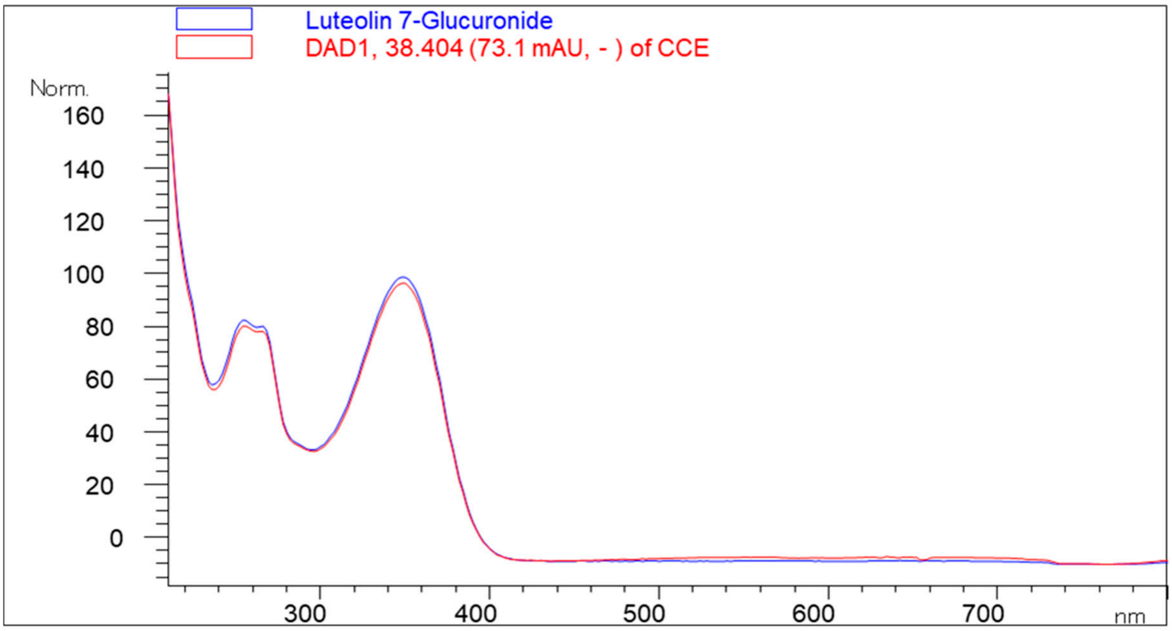

7. 3,4-Dicaffeoylquinic acid

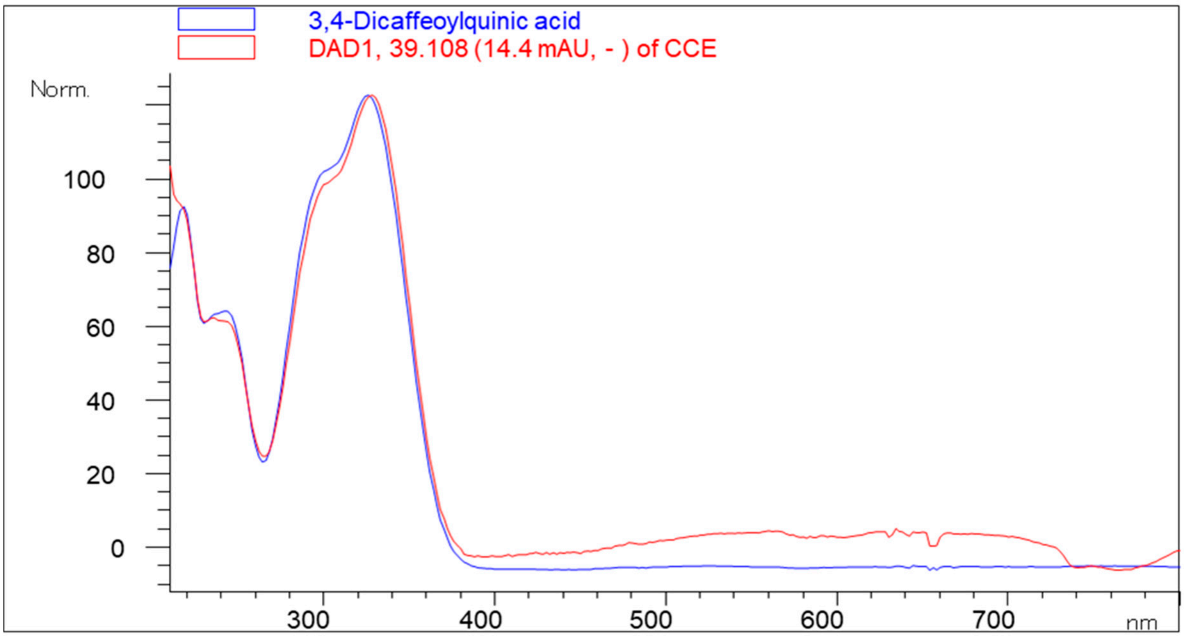

8. Apigenin 7-O-Rutinoside

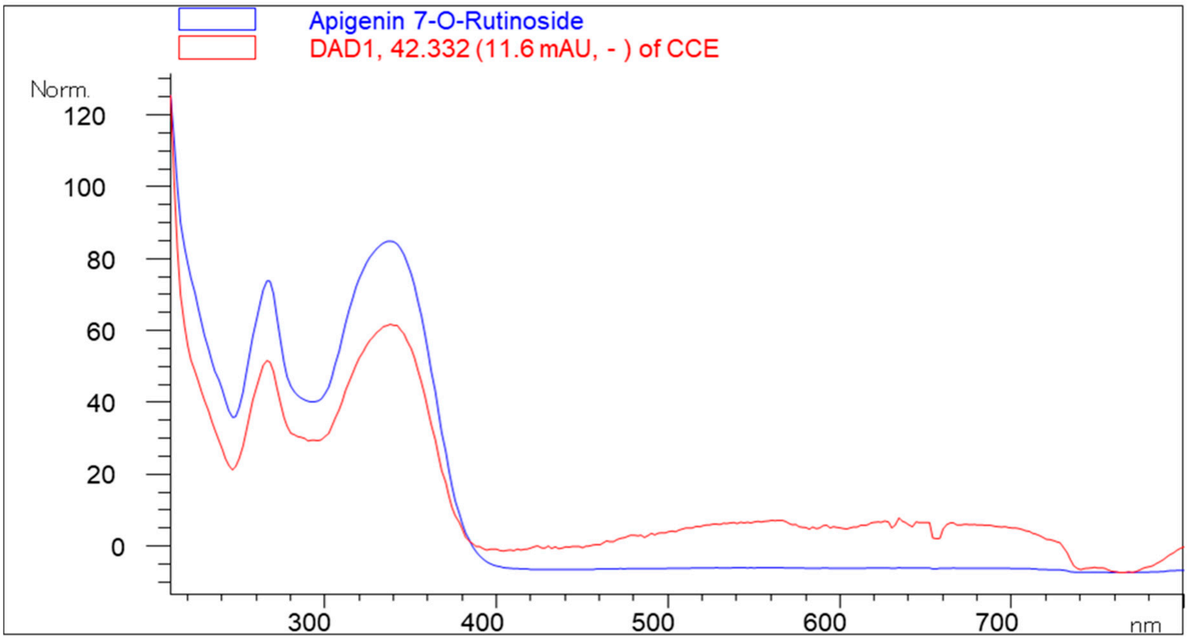

9. 1,5-Dicaffeoulquinic acid

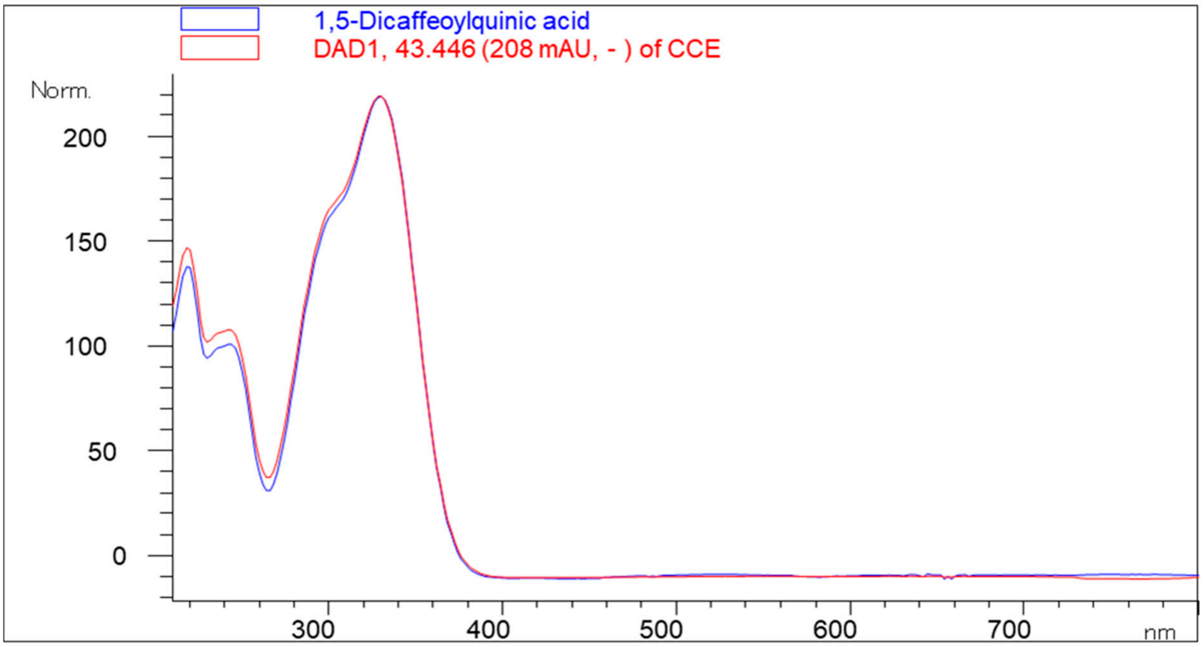

Supplement: Supplementary file 1 [file biology-15-00475-s001.zip › biology-4156049-supplementary.pdf]
