# Supplementary material for: Cynara cardunculus subsp. cardunculus (Wild Artichoke) Extract: Antimicrobial Activity and Cytotoxicity, Apoptosis Induction, and Chemosensitization in Colon Cancer Cells
Source: Biology (Basel). 2026 Mar 15;15(6):475. doi: 10.3390/biology15060475 (PMC13023877; doi:10.3390/biology15060475)
Supplement: Supplementary file 1 [file biology-15-00475-s001.zip › Original WB Images Biology-4156049 .pdf]

Gel 1

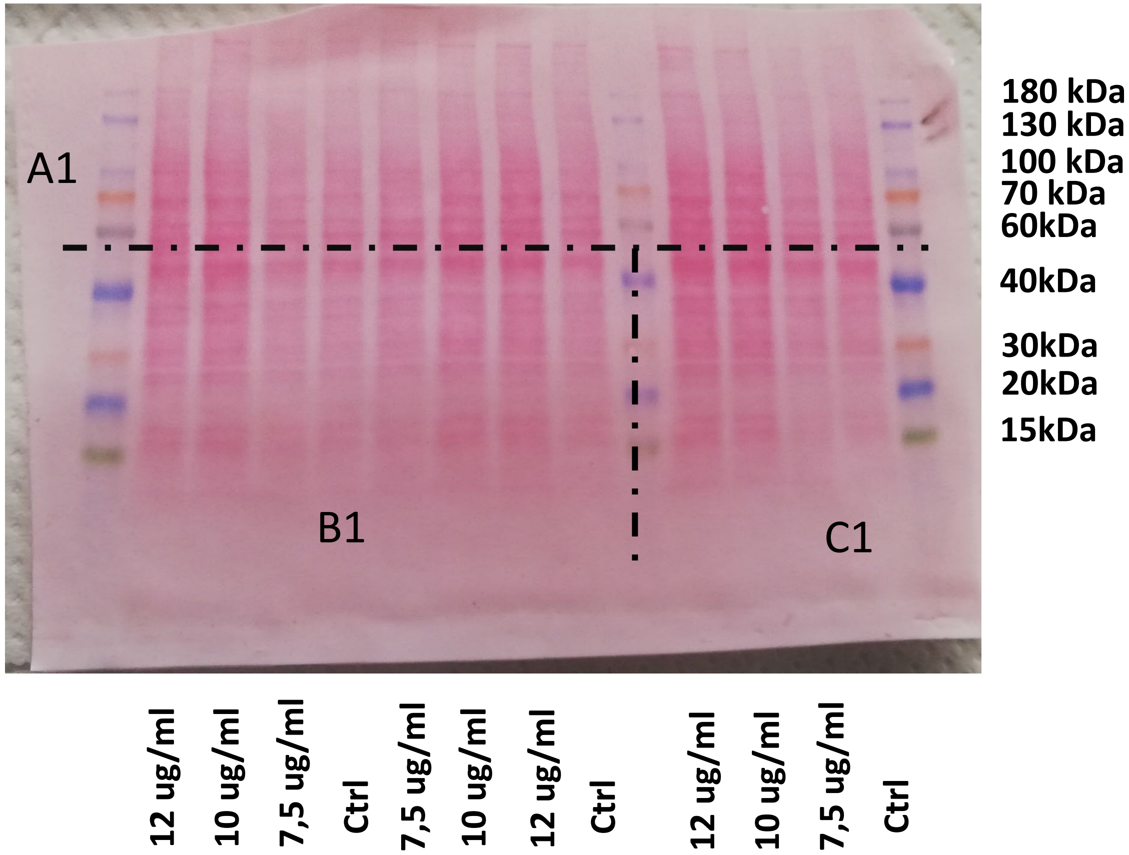

Gel2

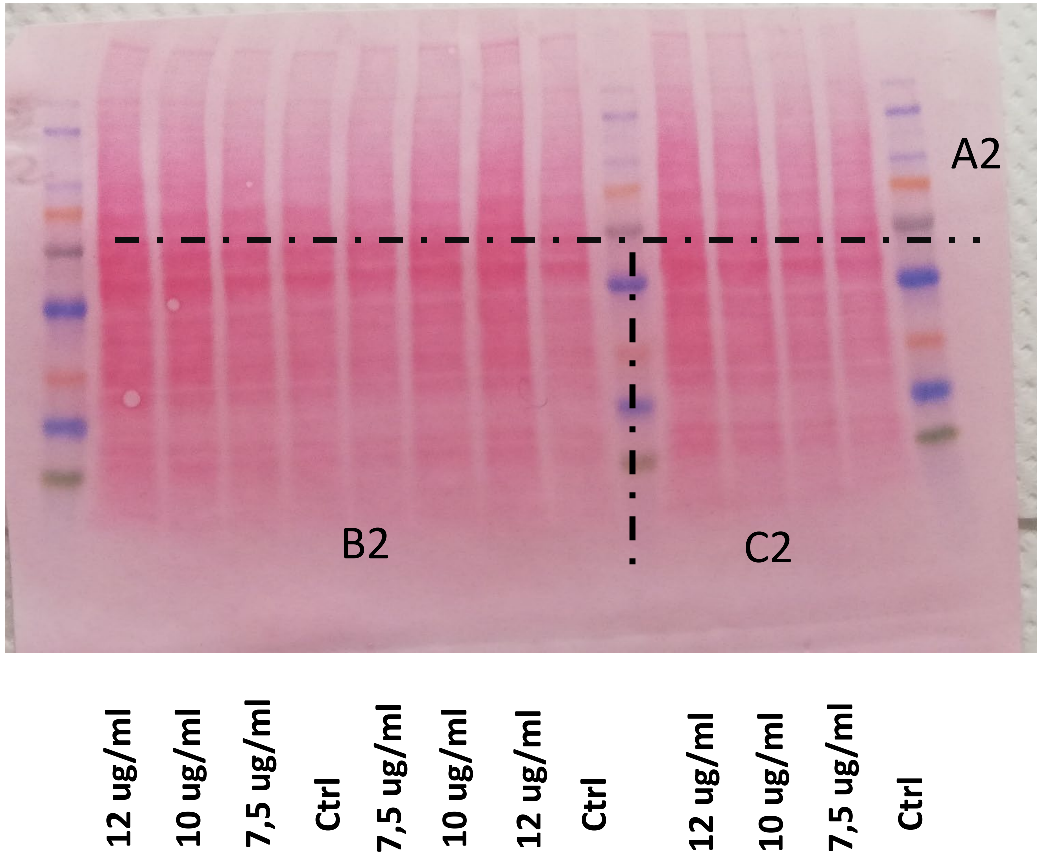

— · — · · The dotted line indicates the cut of memebrane in three pieces for both gels: A superior part; B right part; C left part

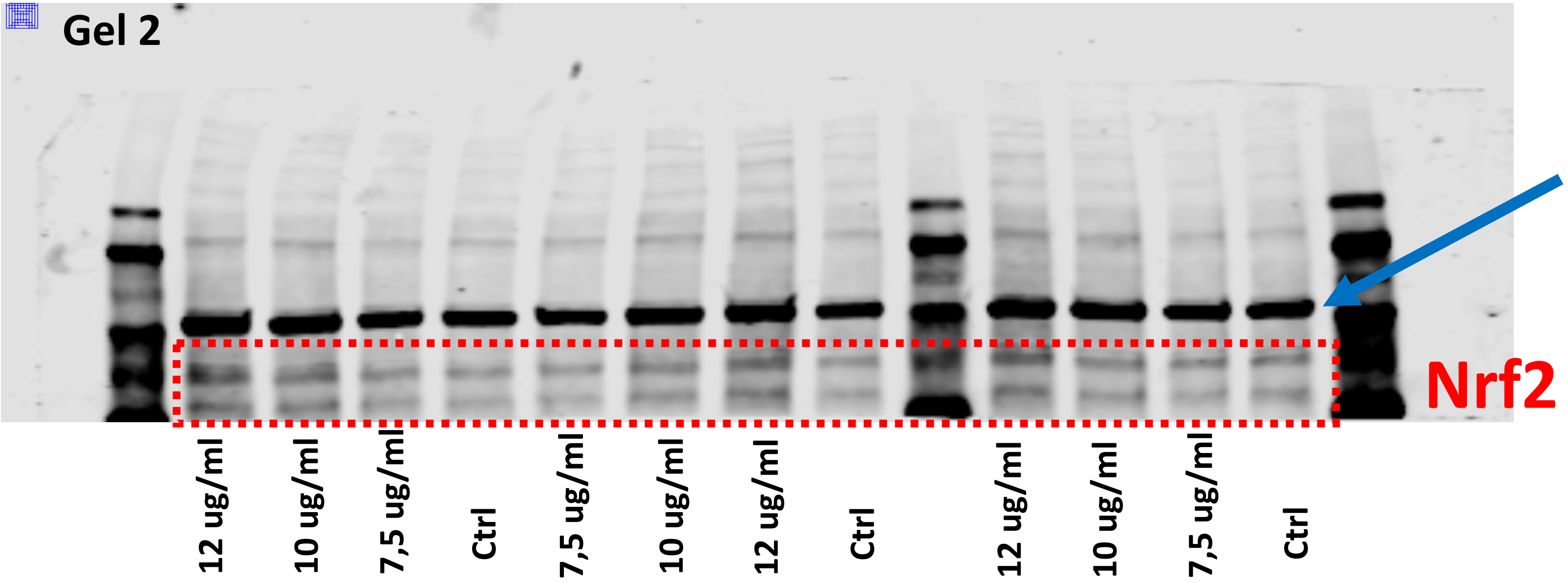

Membrane was colored before Nrf2 with anti-PARP Ab (result is not present in the paper)

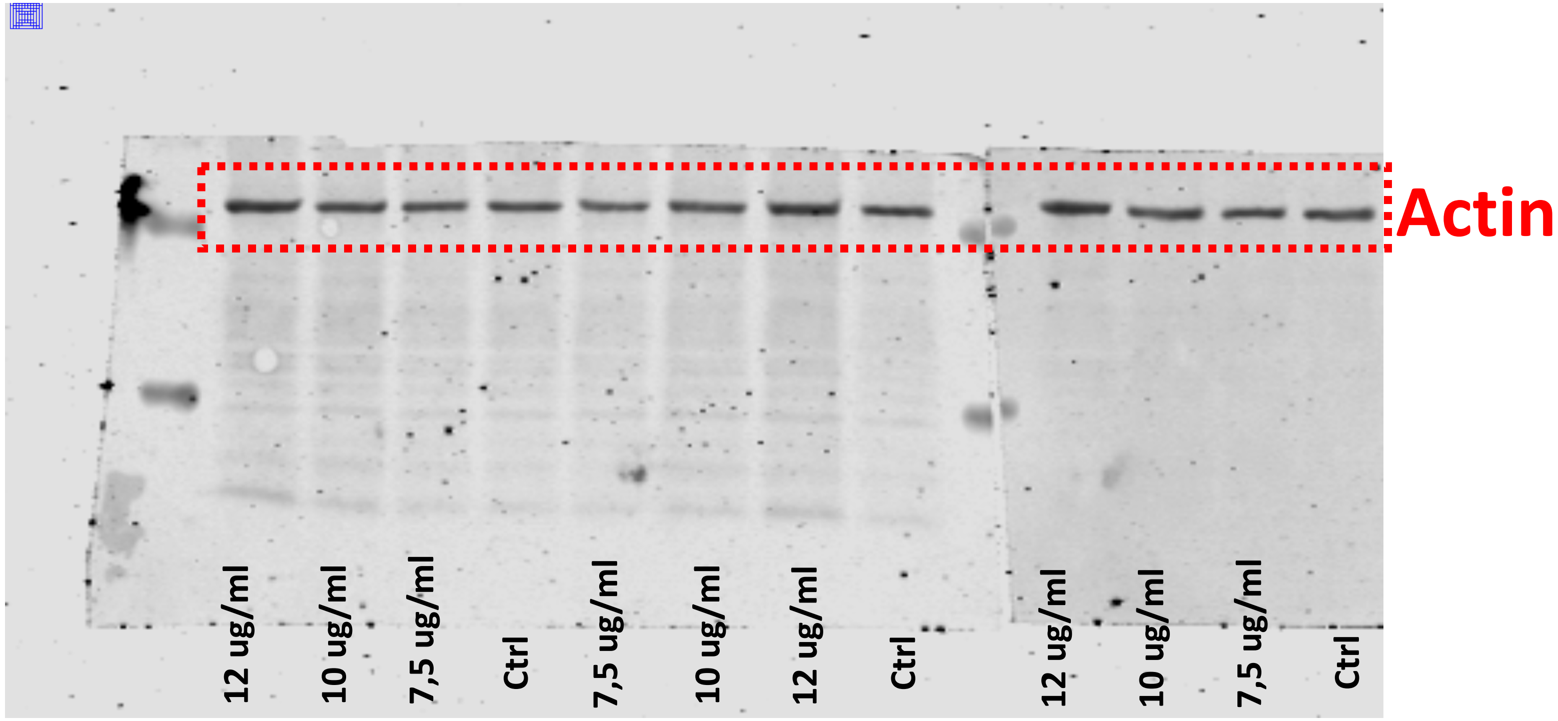

Figure 6

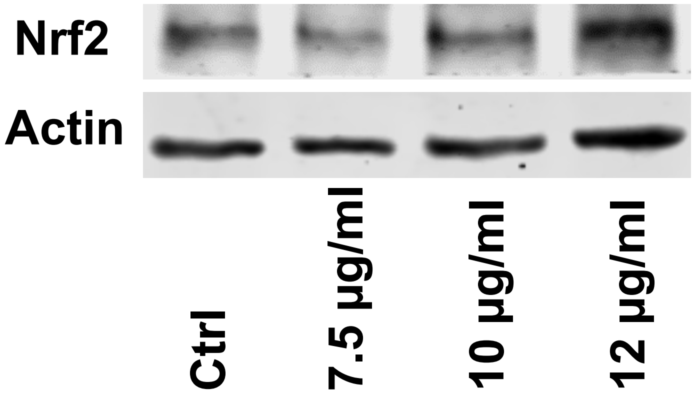

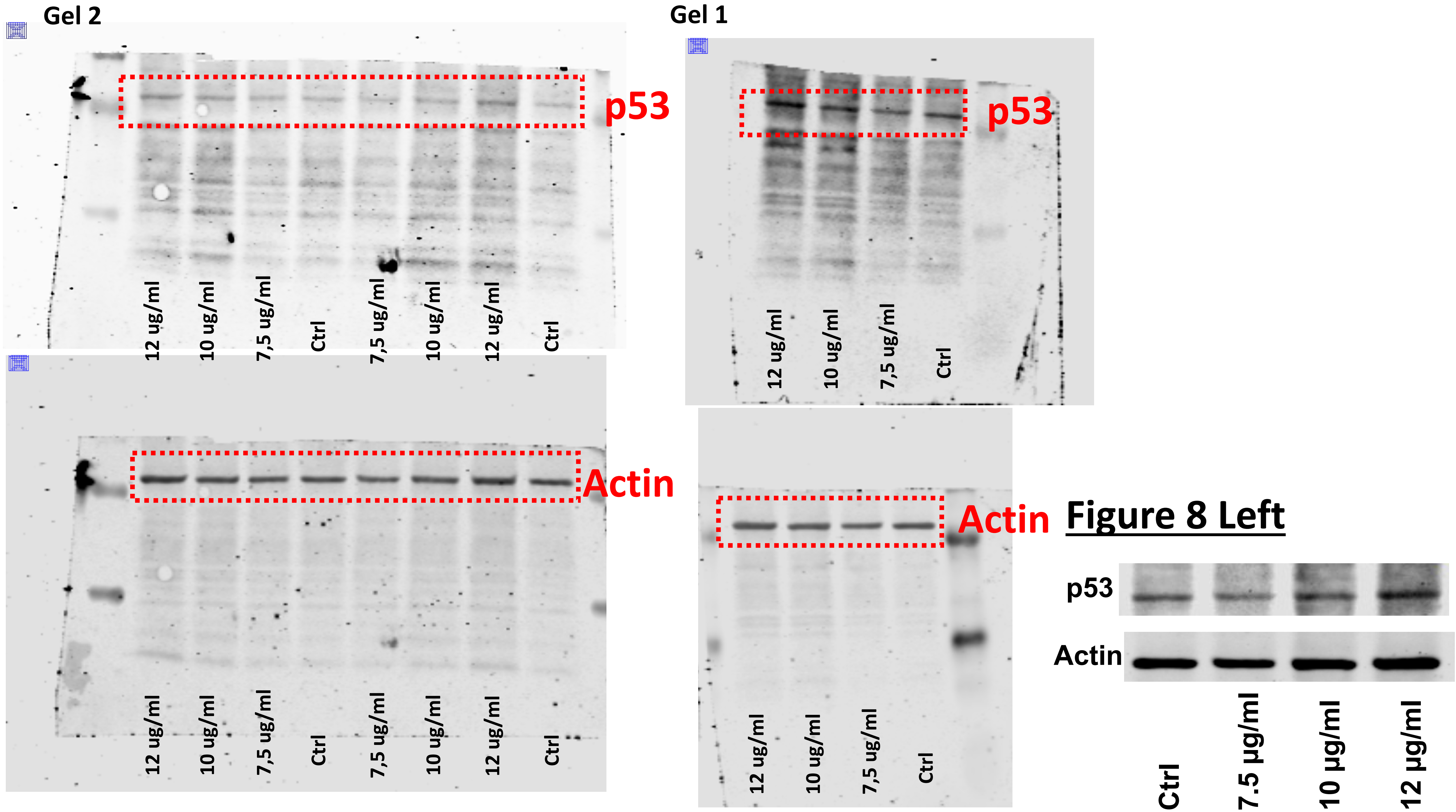

Figure 8 Left

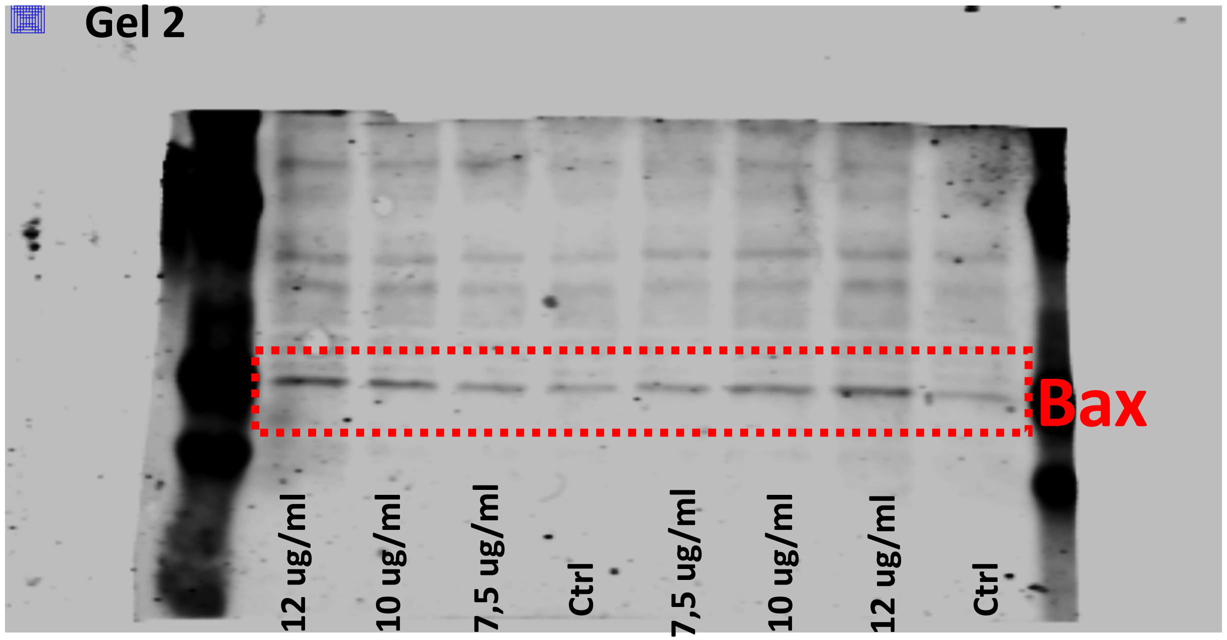

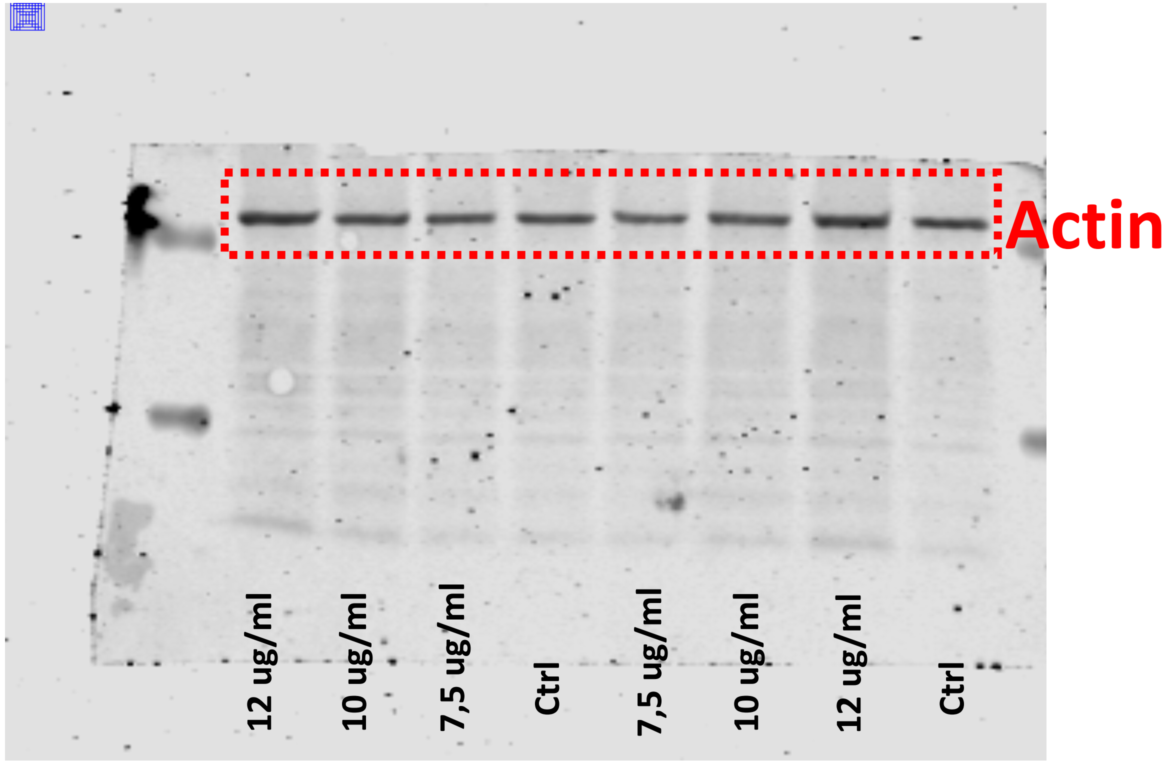

Figure 8 Right

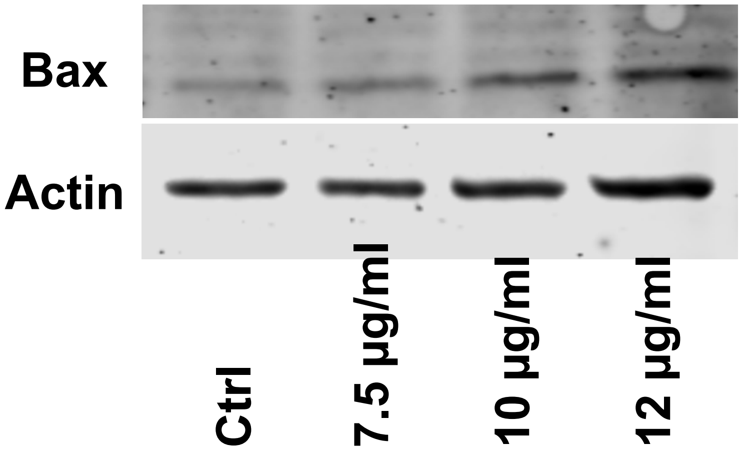

Gel 1

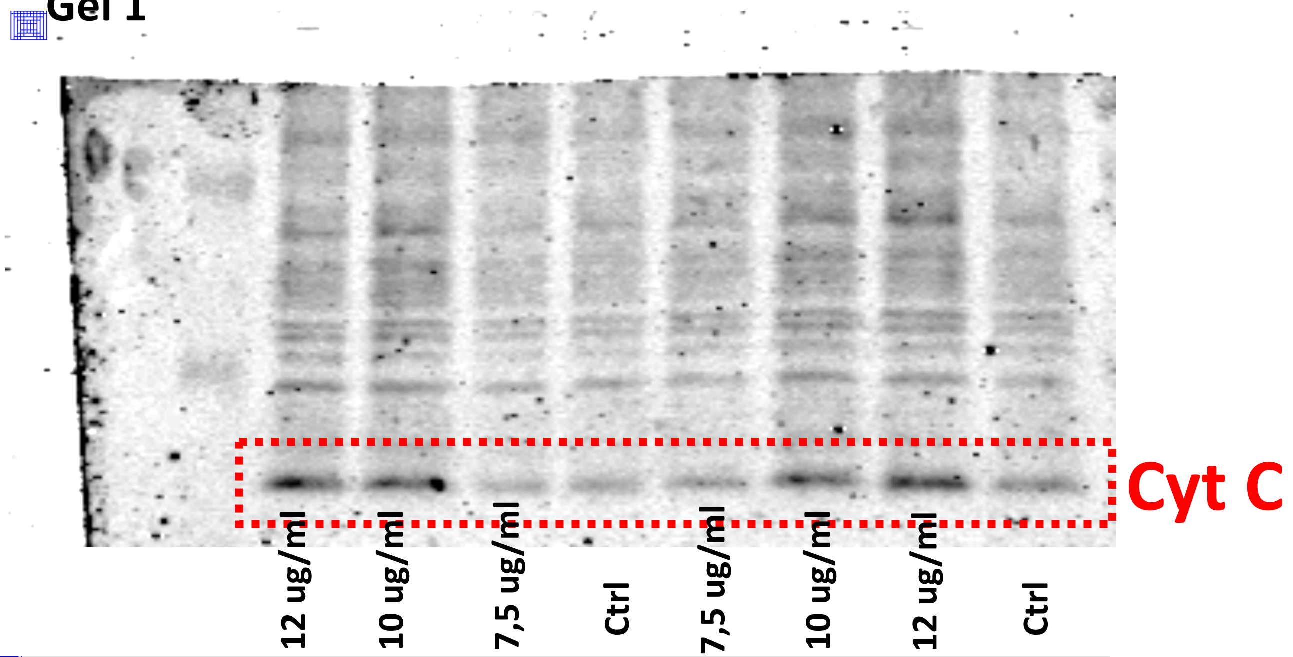

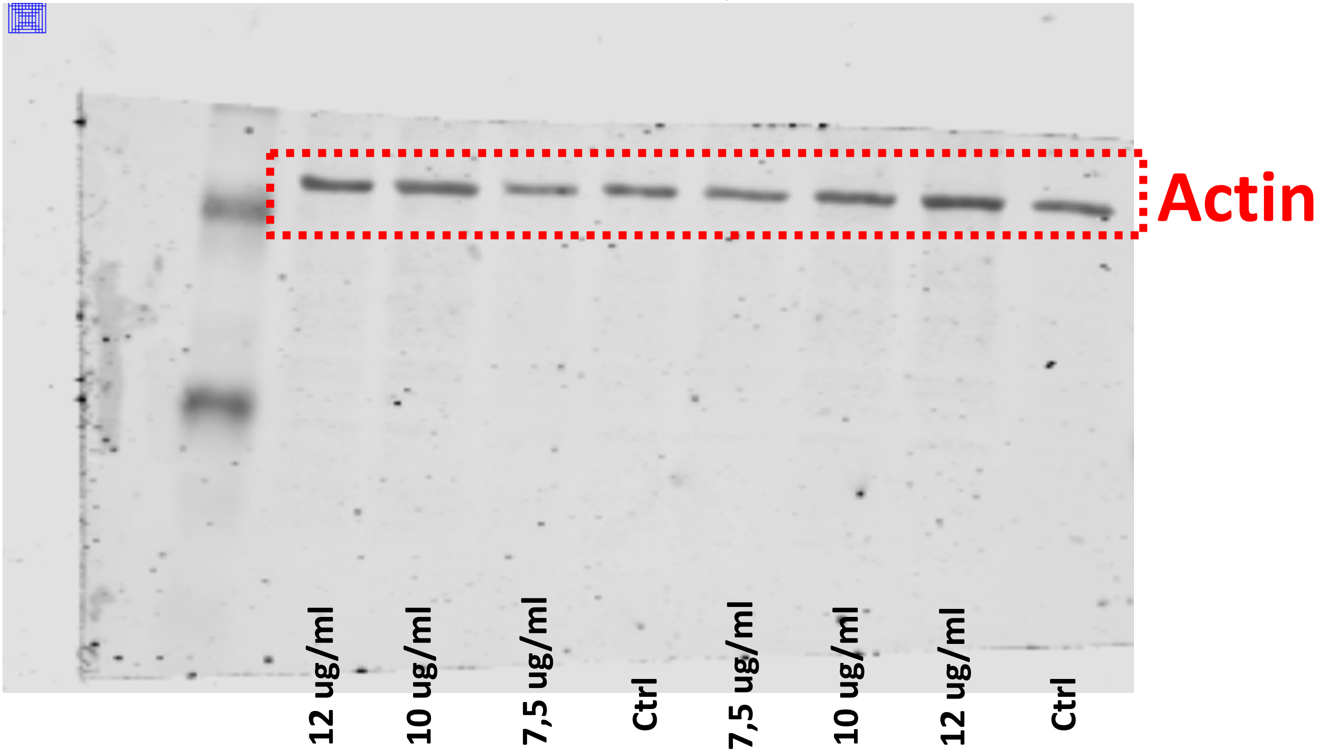

Figure 9 Left

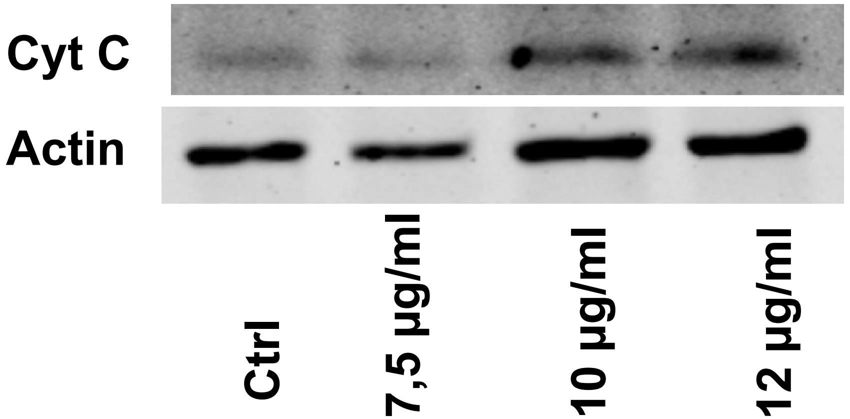

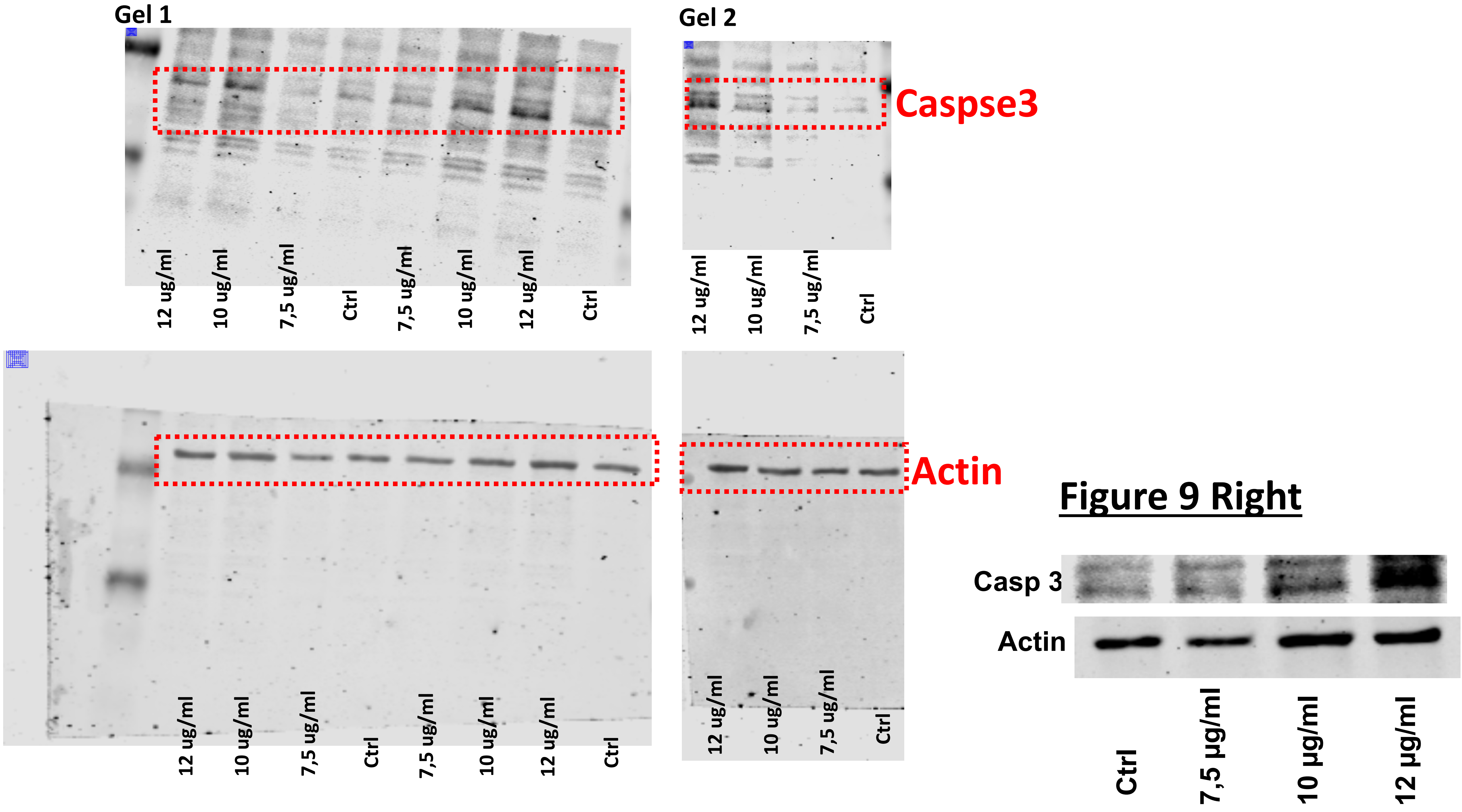
Gel 1
Gel 2
12 ug/ml
10 ug/ml
7,5 ug/ml
Ctrl
7,5 ug/ml
10 ug/ml
12 ug/ml
Ctrl
12 ug/ml
10 ug/ml
7,5 ug/ml
Ctrl
Caspse3
Actin
12 ug/ml
10 ug/ml
7,5 ug/ml
Ctrl
7,5 ug/ml
10 ug/ml
12 ug/ml
Ctrl
12 ug/ml
10 ug/ml
7,5 ug/ml
Ctrl
Figure 9 Right
Casp 3
Actin
Ctrl
7,5 μg/ml
10 μg/ml
12 μg/ml
